# Caspase‐6/Gasdermin C‐Mediated Tumor Cell Pyroptosis Promotes Colorectal Cancer Progression Through CXCL2‐Dependent Recruitment of Myeloid‐Derived Suppressor Cells

**DOI:** 10.1002/advs.202411375

**Published:** 2025-04-11

**Authors:** Hanchao Gao, Yikun Yao, Weilong Li, Zigan Xu, Wenjun Hu, Kewang Luo, Peishan Chen, Wanjing Shang, Shaodong Luan, Guojun Shi, Mengtao Cao, Pengfei Chen

**Affiliations:** ^1^ Department of Nephrology Shenzhen Longhua District Central Hospital Shenzhen Longhua District Key Laboratory for Diagnosis and Treatment of Chronic Kidney Disease Shenzhen Guangdong 518110 China; ^2^ Shanghai Institute of Nutrition & Health Chinese Academy of Science Shanghai 200031 China; ^3^ Department of Anesthesiology The 305 Hospital of Liberation Army of China (PLA) Beijing 100036 China; ^4^ Department of Medical Laboratory People's Hospital of Longhua Shenzhen Guangdong 518110 China; ^5^ Lymphocyte Biology Section Laboratory of Immune System Biology National Institute of Allergy and Infectious Diseases National Institutes of Health Bethesda MD 20892 USA; ^6^ Department of Endocrinology and Metabolism Medical Center for Comprehensive Weight Control The Third Affiliated Hospital of Sun Yat‐sen University Guangzhou 510630 China; ^7^ State Key Laboratory of Oncology in South China Guangzhou 510630 China; ^8^ Department of Respiratory Medicine Shenzhen Longhua District Central Hospital Shenzhen Guangdong 518110 China; ^9^ Department of Traumatic Orthopedics Shenzhen Longhua District Central Hospital Shenzhen Guangdong 518110 China

**Keywords:** colorectal cancer, CXCL2, GSDMC, HMGB1, myeloid‐derived suppressor cells

## Abstract

Gasdermin (GSDM) family proteins mediate inflammatory cell pyroptosis and exert critical contributions to the pathogenesis of gastrointestinal cancers, infections, and gut mucosal inflammation. Gasdermin C (GSDMC) is overexpressed in human colorectal cancer (CRC); however, the molecular mechanisms underlying GSDMC regulation of CRC tumorigenesis are largely elusive. Here, it is found that both GSDMC expression and activation are significantly elevated in human and mouse CRC tissues. *Gsdmc2/3/4* deficiency attenuates tumor progression in both chemically induced CRC mouse model and spontaneous intestinal tumor model. Mechanistically, under hypoxia and low‐glucose condition, GSDMC2/3/4 are directly activated by Caspase‐6, but not by Caspase‐8, as previously reported in other cancers. GSDMC2/3/4‐mediated pyroptosis in tumor cells leads to the release of high mobility group protein B1 (HMGB1), which enhances the expression of chemokine attractant C‐X‐C motif chemokine 2 (CXCL2) in surrounding tumor cells. Subsequently, the elevated CXCL2 secretion from tumor cells promotes the recruitment of myeloid‐derived suppressor cells (MDSCs) into the tumor microenvironment (TME) through C‐X‐C chemokine receptor type 2 (CXCR2), thereby facilitating CRC progression. These findings reveal a mechanism by which Caspase‐6/GSDMC‐mediated tumor cell pyroptosis, in response to hypoxic and low‐glucose conditions, remodels the immunosuppressive microenvironment through CXCL2‐dependent recruitment of MDSCs. These results identify GSDMC as a potential drug target for CRC therapy.

## Graphical abstract

Hypoxic and low‐glucose conditions in tumor tissues trigger colorectal tumor cell pyroptosis via sequential activation of the Caspase‐1/Caspase‐6/GSDMC axis. High mobility group box 1 (HMGB1), released from GSDMC‐mediated pyroptotic tumor cells, upregulates CXCL2 expression in adjacent tumor cells, thereby recruiting myeloid‐derived suppressor cells (MDSCs) into the tumor microenvironment (TME) to promote colorectal cancer progression.

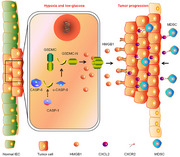

## 1 Introduction

Colorectal cancer (CRC) is the third most prevalent malignancy and the second leading cause of cancer‐related deaths worldwide.^[^ ^1^, ^2^ ^]^ From the perspective of molecular mechanisms underlying carcinogenesis of CRC, genetic hypermutability accounts for 70% of cases, including mutations in Adenomatous polyposis coli (*APC*), Kirsten rat sarcoma viral oncogene homolog (*KRAS*), Phosphatidylinositol‐4,5‐bisphosphate 3‐kinase catalytic subunit alpha (*PIK3CA*), B‐Raf proto‐oncogene (*BRAF*), SMAD family member 4 (*SMAD4*), or Tumor protein p53 (*TP53*).^[^ ^3^, ^4^ ^]^ However, tumor development largely depends on the close interaction of mutant cells with their tumor microenvironment (TME). The TME is primarily composed of three cell types including tumor‐associated fibroblasts, intratumor vascular cells, and infiltrating immune cells. The tumor infiltrating immune cells comprise cytotoxic T cells, regulatory T cells, T_H_2 cells, T_H_17 cells, macrophages, natural killer cells, myeloid‐derived suppressor cells (MDSCs), and B cells, which exhibit diverse functionalities in restricting or driving tumor progression.^[^ ^3^ ^]^ MDSCs are a cluster of tumor infiltrating immune cells in TME, which can indirectly suppress anti‐tumor immune responses or directly promote tumor cell survival, proliferation, and metastasis.^[^ ^5^ ^]^

The imbalance between rapidly proliferating tumor cells and blood supply often leads to hypoxia and low‐glucose condition of TME.^[^ ^6^ ^]^ Hypoxia is a well‐known feature of solid tumors and an independent prognostic indicator, which is related to poor survival in various cancer types including intestinal cancer.^[^ ^7^ ^]^ The hypoxic and low‐glucose TME contributes to immunosuppression by modulating tumor cell metabolism, intercellular communication, epi‐transcriptomic, and epigenomic regulation.^[^ ^8^ ^]^ Hypoxia has been demonstrated to be required for the differentiation and function of MDSCs in hepatocellular carcinoma and melanoma.^[^ ^9^, ^10^, ^11^ ^]^ However, the detailed mechanisms of hypoxia and low‐glucose TME that regulate the recruitment function of MDSCs in CRC development remain largely unclear.

Since the discovery of Gasdermin (GSDM) family of proteins as the final executors of pyroptosis,^[^ ^12^ ^]^ a growing body of evidence has demonstrated the roles of GSDM‐mediated pyroptosis in inflammatory diseases, cancer, and hematopoietic disorders.^[^ ^13^ ^]^ The GSDM family includes six members: *GSDMA/B/C/D/E* and *Pejvakin* (*PJVK*).^[^ ^14^ ^]^ There is one *GSDMC* gene in humans and four orthologs in mice (*Gsdmc1/2/3/4*). GSDMC is highly expressed in several cancers and correlates with the poor survival rate.^[^ ^15^, ^16^, ^17^ ^]^ It was reported that α‐ketoglutarate induces the intracellular DR6‐containing receptosome for activation of GSDMC by Caspase‐8 in cancer cell lines.^[^ ^18^ ^]^ Under hypoxia condition, phosphorylated signal transducer and activator of transcription 3 (p‐STAT3) forms a complex with nuclear PD‐L1 to activate the transcription of GSDMC in breast cancer cells and increases GSDMC‐mediated cell pyroptosis to promote tumor necrosis.^[^ ^16^ ^]^ T cell derived granzyme B activates Caspase‐6, which subsequently cleaves GSDMC to induce pyroptosis, sensitizing breast cancer cells to poly (ADP‐ribose) polymerase (PARP) inhibitor therapy.^[^ ^19^ ^]^ Beyond its role in cancer, GSDMC plays vital roles in intestinal epithelial cell (IEC) pyroptosis during type 2 immune response, facilitating the release of antiparasitic factors or IL‐33 to induce intestinal inflammation.^[^ ^20^, ^21^ ^]^ In the pathogenesis of intestinal cancer, the GSDM family proteins might play different roles in CRC development.^[^ ^17^ ^]^ Specifically, GSDMC2 and GSDMC4 were upregulated by the inactivation of TGFβR Type II in the *Apc* mutation mouse model, and GSDMC promoted colon cancer cells proliferation and colony formation in vitro.^[^ ^22^ ^]^ However, it is unclear whether GSDMC is involved in the regulation of intestinal cancer development.

Here, we analyzed the expression levels and activation of GSDMC in human CRC tissue‐array and mouse CRC tissues and revealed the elevated expression and activation of GSDMC in CRC tissues. We demonstrated that *Gsdmc2/3/4* deficiency in mice attenuated intestinal tumor development in both chemically induced CRC model and *Apc* ^min/+^ spontaneous intestinal tumor model. Mechanistically, we revealed that GSDMC was activated by Caspase‐6 in intestinal tumors under hypoxia and low‐glucose condition. The activation of GSDMC in tumor cells promoted tumor progression through facilitating the release of HMGB1 and CXCL2‐dependent recruitment of MDSCs to orchestrate the immunosuppressive microenvironment.

## 2 Results

### 2.1 GSDMC is Elevated and Activated in Intestinal Cancer

**Figure 1:**
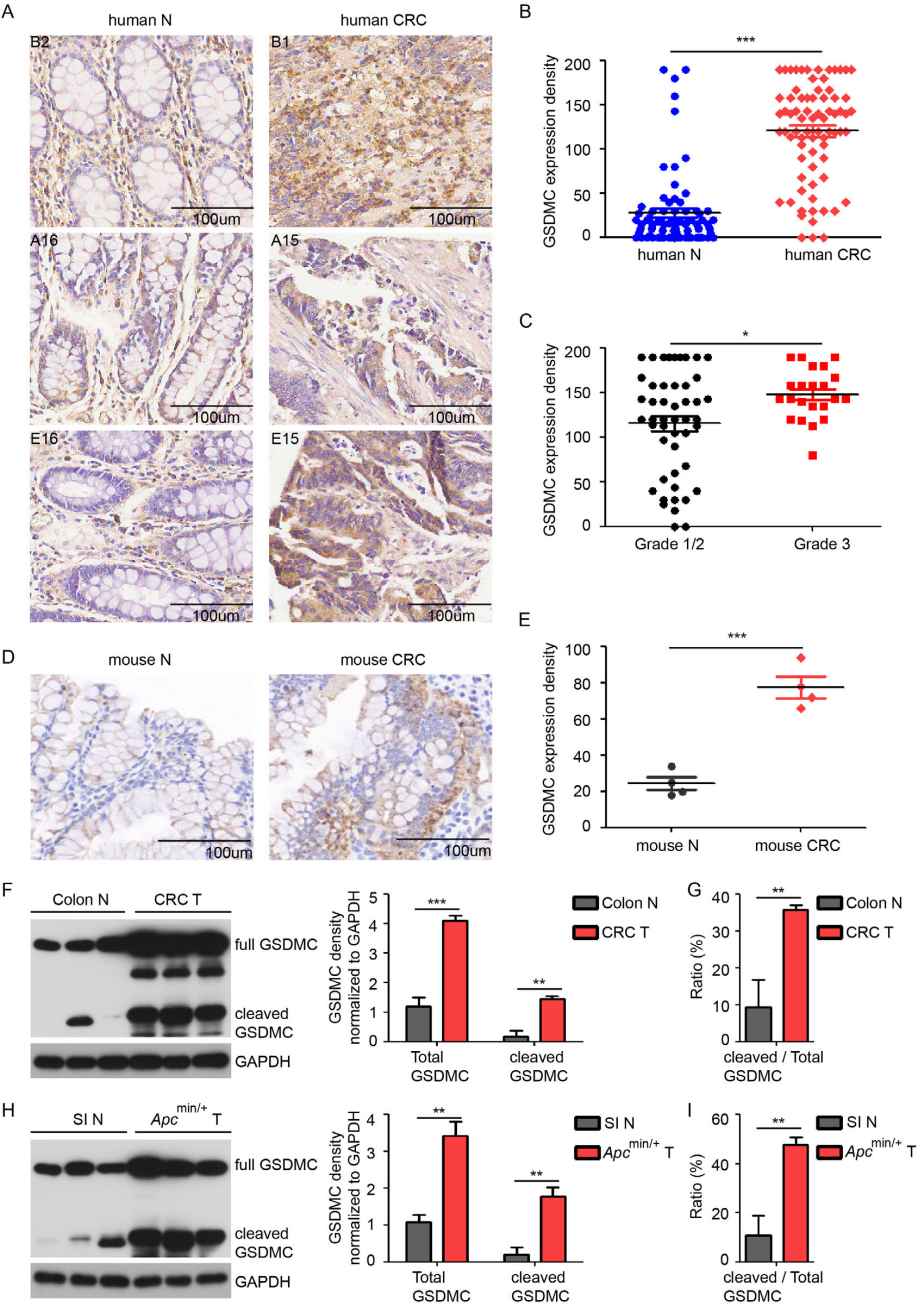
GSDMC is induced and activated in intestinal cancer. A) Representative images showing GSDMC staining in human CRC microarray. B,C) Quantification of GSDMC expression density in human CRC microarray. D) Immunohistochemical staining of GSDMC in AOM‐DSS induced mouse CRC and normal colon tissue. E) Quantification of GSDMC expression density in (D). F–I) Western blot analysis of GSDMC expression in mouse CRC, normal colon (F), and in intestinal tumors from *Apc^min/+^* mice and small intestine (H). Bar graphs represent quantitative analysis of total GSDMC (full length plus cleaved) and cleaved GSDMC, normalized to GAPDH band intensity. The ratio of cleaved N‐terminal GSDMC to total GSDMC (G,I). Data are representative of at least three independent experiments (mean ± SEM in [B,C,E–I]). *p < 0.05, **p < 0.01, and ***p < 0.001 by Student's *t*‐test. SI: small intestine, N: normal colon.

We first analyzed the transcriptional expression of GSDM family members in colorectal adenocarcinoma using The Cancer Genome Atlas (TCGA) dataset. We found that the transcriptional levels of GSDMA, GSDMC, and PJVK were significantly increased in human colorectal tumors; while, the expression of GSDMB, GSDMD, and GSDME remained unchanged. Among the GSDM family members, GSDMC showed the most remarkable upregulation (≈tenfold change) in human colorectal tumors (Figure S1A, Supporting Information). In addition, the median expression levels of GSDMC were significantly higher in tumor tissues for most of the cancers analyzed (13/17) (Figure S1B, Supporting Information). To understand the role of GSDMC in regulating CRC development, we first determined the GSDMC protein level by immunohistochemical assay of a human tissue‐array, which included colorectal tumors and paired non‐tumor tissues from 75 patients. Compared to adjacent non‐tumor tissues, the expression of GSDMC was significantly higher in colorectal cancers (**Figure** **1**A,B; Figure S1C, Supporting Information). High GSDMC expression was observed in 95% (21/22) of grade 3 CRC cases and 68% (34/50) of grade 1/2 CRC cases, suggesting a potential positive correlation between GSDMC expression levels and tumor progression (Figure 1C). Although the transcript levels of GSDMC were similar among subgroups by fractional analysis according to tumor stage, metastasis, subtype, and age, its expression was significantly higher in colon adenocarcinoma than in normal tissues (Figure S1D, Supporting Information). A decreased survival rate in patients with colon adenocarcinoma was associated with higher levels of GSDMC expression (Figure S1E, Supporting Information) from the TCGA dataset.

Further, GSDMC expression was also upregulated in the Azoxymethane‐Dextran sulfate sodium (AOM‐DSS) induced CRC mouse model (Figure 1D,E). Increased expression and activation of GSDMC, as evidenced by the generation of its N‐terminal fragment, were detected in CRC tissues by Western blotting (Figure 1F). The significantly increased ratio of N‐terminal fragment to total GSDMC was observed in CRC tissues, indicating enhanced activation of GSDMC beyond increased expression (Figure 1G). Similarly, there was an increase in the expression and activation of GSDMC in spontaneous intestinal tumors from *Apc* ^min/+^ mice compared with normal intestinal tissue (Figure 1H,I). In addition, we determined the mRNA expression levels of GSDMC orthologs by qPCR in mouse intestinal tumors. GSDMC2/3/4 showed an upregulation of more than tenfold in mouse CRC compared to normal colon; while, GSDMC1 remained unchanged (Figure S1F, Supporting Information). GSDMC1/2/3/4 were increased in intestinal tumors from *Apc* ^min/+^ mice (Figure S1G, Supporting Information). Taken together, our data demonstrate that the expression and activation of GSDMC are significantly increased in intestinal tumors.

### 2.2 GSDMC2/3/4 Promote AOM‐DSS Induced Colorectal Cancer Progression

**Figure 2:**
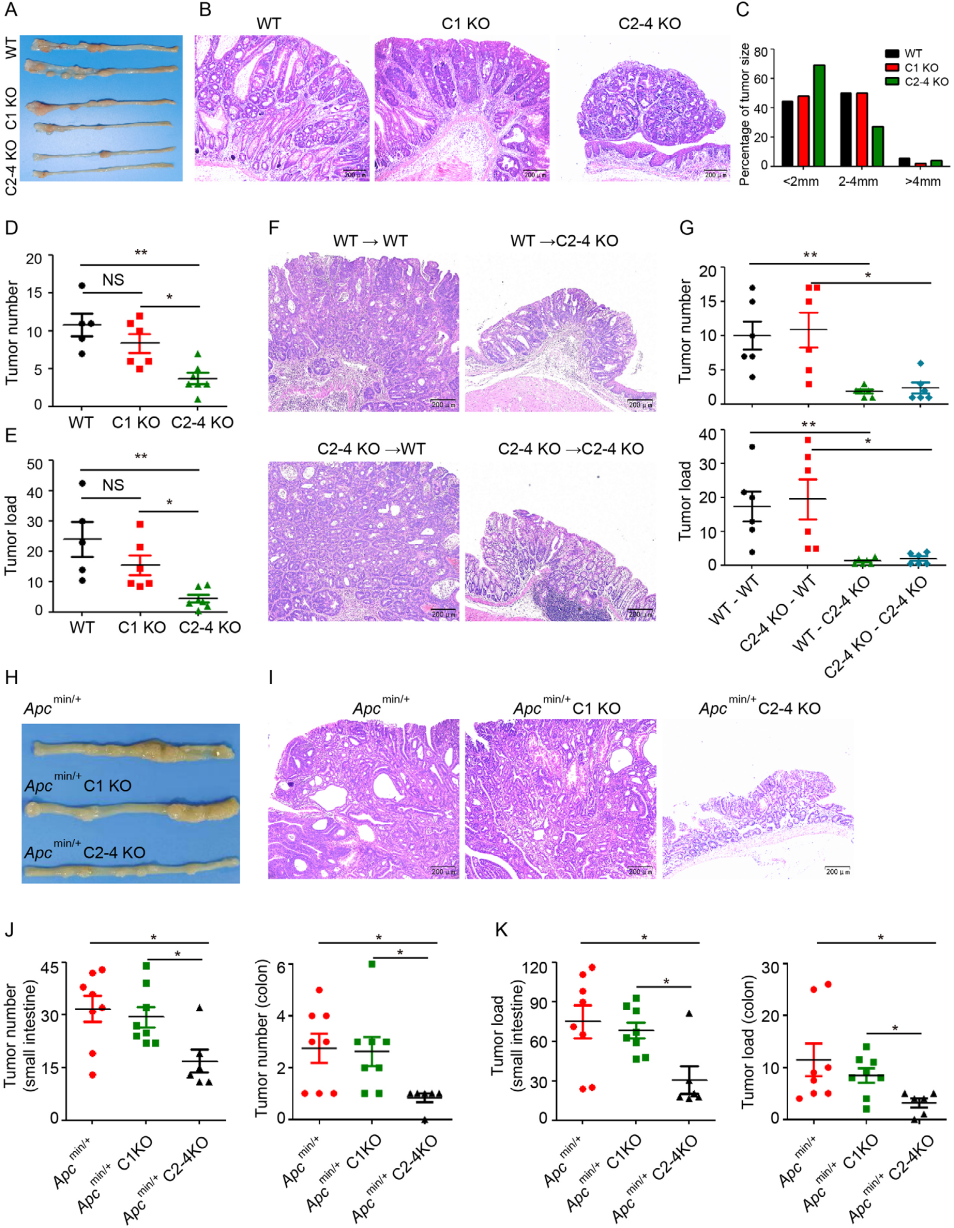
GSDMC2/3/4 promote intestinal cancer progression. A,B) Macroscopic view (A) and H&E histology (B) of the representative colons from WT, *C1* KO, and *C2–4* KO mice at the end of the AOM‐DSS model. C) Histogram showing the size distribution of tumors as indicated in (A). D,E) Tumor number (D) and tumor load (E) from mice as indicated in (A). F) H&E histology of the representative tumors from the BM‐transplanted mice at the end of the AOM‐DSS model. G) Colon tumor number and tumor load from mice as indicated in (F). H,I) Macroscopic view (H) and H&E histology (I) of the representative small intestines from 20‐week old *Apc^min/+^*, *Apc^min/+^ C1* KO, and *Apc^min/+^ C2‐4* KO mice. J,K) Tumor number (J) and tumor load (K) in the small intestines or colons from spontaneous intestinal tumor model in (H). Data are representative of two (F,G) or three (A–E) independent experiments (mean ± SEM in [D, E, G, J and K]). *p < 0.05 and **p < 0.01 by Student's *t*‐test. NS: no significance.

To study the role of GSDMC in tumor pathogenesis, we first assessed the expression of four GSDMC orthologs in mouse tissues and found abundant mRNA expression of GSDMC2, GSDMC3, and GSDMC4 in colon tissue; while, GSDMC1 mRNA expression was relatively low (Figure S2A, Supporting Information). Given the increased expression and activation of GSDMC in colorectal cancer, we next asked whether GSDMCs played a vital role in CRC development. We generated *Gsdmc1* knockout mice (*C1* KO, *Gsdmc1* ^−/−^) and *Gsdmc2/3/4* triple knockout mice (*C2‐4* KO, *Gsdmc2‐4* ^−/−^) and induced CRC development using the AOM‐DSS model. Deficiency of *Gsdmc1* had a marginal effect on tumor formation; while, deficiency of *Gsdmc2‐4* dramatically reduced colon tumor formation (**Figure** **2**A). The limited effect of GSDMC1 on CRC development could be explained by its relatively low transcriptional abundance in mouse colon (Figure S2A, Supporting Information). H&E staining revealed decreased tumor size in *Gsdmc2‐4* ^−/−^ mice (Figure 2B). Statistical analysis of all visible tumors revealed that most of the tumors in *Gsdmc2‐4* ^−/−^ mice were small (<2 mm); while, wild type littermates and *Gsdmc1* ^−/−^ mice developed more tumors with large size (2–4 mm) (Figure 2C). Both tumor number and tumor load were significantly diminished in *Gsdmc2–4* ^−/−^ mice (Figure 2D,E). Consistent with repressed tumor progression, *Gsdmc2–4* deficiency alleviated body weight loss and promoted recovery in the latter half of the experiment schedule (Figure S2B, Supporting Information). As developed tumors can elicit inflammation, *Gsdmc2–4* deficiency also decreased expression of proinflammatory genes (Figure S2C, Supporting Information). Taken together, our data suggest that GSDMC2–4, but not GSDMC1, are involved in promoting CRC progression.

### 2.3 Epithelial Cell‐Derived GSDMC2/3/4 Promote AOM‐DSS Induced Colorectal Cancer Development

We next characterized the cell type‐specific expression pattern of GSDMC within colon tissues and found that GSDMC expression was restricted to CDH1^+^ epithelial cells, but not in CD45^+^ hematopoietic cells or COL3A1^+^ fibroblast cells, based on single‐cell RNA sequencing data (Figure S3A, Supporting Information). Multicolor immunofluorescence staining confirmed that the E‐cadherin^+^ epithelial cells expressed GSDMC in both the colon and small intestine (Figure S3B, Supporting Information). These data suggest that GSDMC is specifically expressed in intestinal epithelial cells. To explore the contribution of intestinal epithelial cells versus immune cells in GSDMC‐driven colorectal tumor development, bone marrow transplantation followed by AOM‐DSS challenge was performed. Irradiated wild‐type (WT) recipients reconstituted with WT or *Gsdmc2–4* ^−/−^ bone marrow cells showed comparable tumor size (Figure 2F, left panel), tumor number, and tumor load (Figure 2G), suggesting that GSDMC in immune cells is dispensable for AOM‐DSS induced CRC progression. Meanwhile, reconstituted *Gsdmc2–4* ^−/−^ recipient mice showed reduced tumor size (Figure 2F, right panel), tumor number, and tumor load (Figure 2G), regardless of the donor by comparison to reconstituted WT recipient mice. These results demonstrate that GSDMC2–4 in intestinal epithelial cells are critical for colorectal cancer progression in this mouse model.

The AOM‐DSS induced CRC mouse model is typically correlated with colitis. Thus, we asked whether GSDMC was involved in acute colitis development. Interestingly, *Gsdmc2–4* deficiency protected mice from DSS‐induced colitis (Figure S4A,B, Supporting Information). Consistently, *Gsdmc2–4* deficiency led to decreased inflammatory cell infiltration and less severe disruption of the mucosal epithelium (Figure S4C, Supporting Information), and protected mice from DSS‐induced cell death (Figure S4D, Supporting Information). These data suggest that GSDMC2/3/4 promote DSS‐induced colitis.

### 2.4 GSDMC2/3/4 Promote Spontaneous Intestinal Cancer Progression

To determine whether GSDMC functions in promoting spontaneous intestinal tumor development, we generated *Apc* ^min/+^ *Gsdmc1* ^−/−^ and *Apc* ^min/+^ *Gsdmc2–4* ^−/−^ mice. Deficiency of *Gsdmc2–4* in *Apc* ^min/+^ mice dramatically suppressed spontaneous intestinal tumor development (Figure 2H,I). Both tumor number (Figure 2J) and tumor load (Figure 2K) were significantly reduced in the small intestine and colon. Similar to the AOM‐DSS induced CRC, *Apc* ^min/+^ *Gsdmc2–4* ^−/−^ mice developed smaller tumors (Figure S5A, Supporting Information). Consistent with the less severe tumor development, the anemia and thymus atrophy in *Apc* ^min/+^ mice were significantly ameliorated by *Gsdmc2–4* deficiency (Figure S5B,C, Supporting Information). Taken together, our data demonstrate that GSDMC2–4 play a vital role in spontaneous intestinal tumor development.

### 2.5 GSDMC2/3/4 Promote Colorectal Tumor Cell Proliferation In Vivo

**Figure 3:**
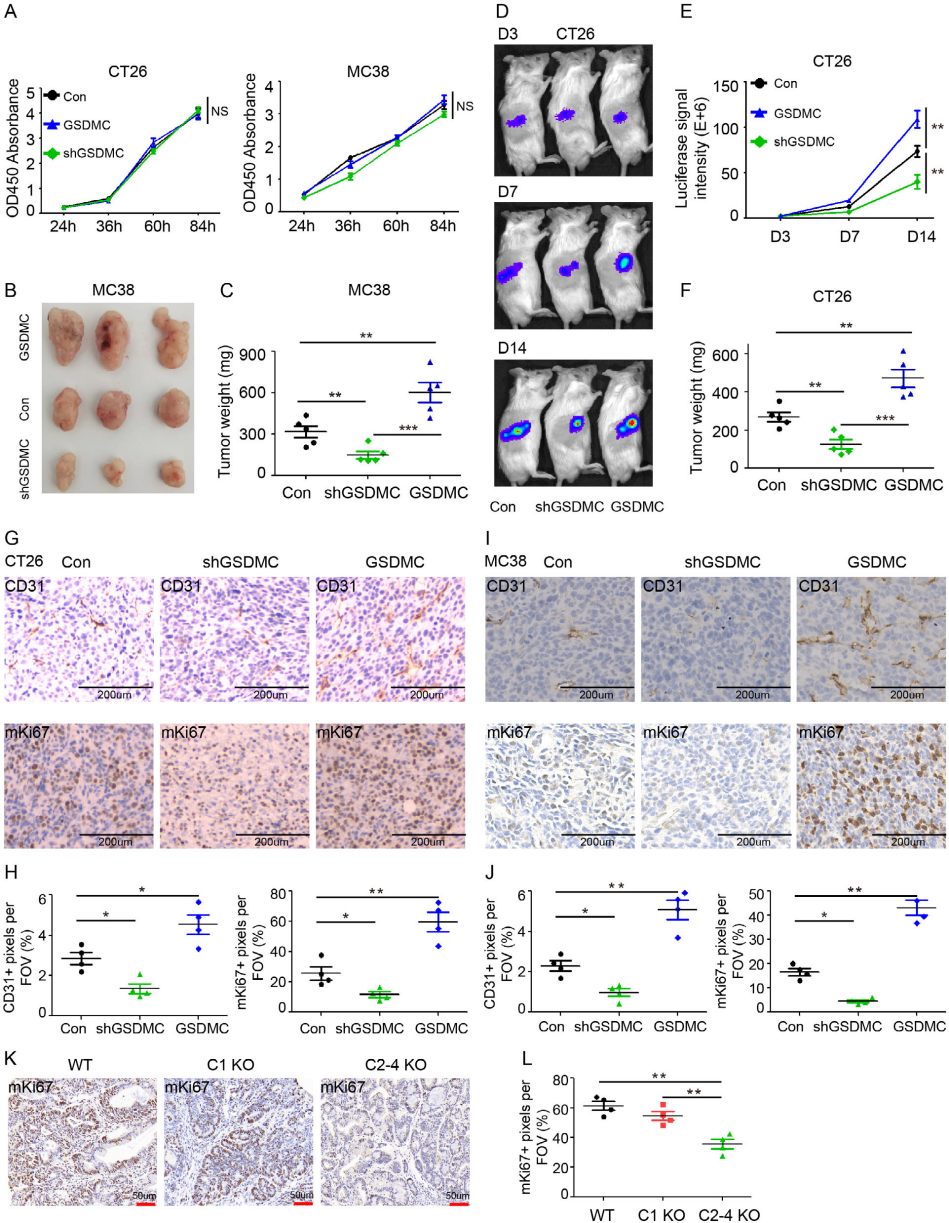
GSDMC2/3/4 promote tumor proliferation in vivo. A) In vitro cell proliferation was assessed using CCK‐8 assay. B,C) Macroscopic view of the representative tumors (B) and tumor weight (C) of MC38 cells stably expressing shGSDMC, GSDMC2‐4, or control empty vectors in the subcutaneous tumor graft model. D–F) Representative bioluminescence images (D), tumor growth curves (E), and tumor weight (F) of CT26 cells stably expressing shGSDMC, GSDMC2‐4, or control empty vectors in the subcutaneous graft model. G–J) CT26 tumors (G,H) or MC38 tumors (I,J) were sectioned and subjected to immunohistochemical staining of CD31 (upper panel) and Ki67 (lower panel). Scatter plots represent the percentages of positive staining signals per field of view (FOV). K) Microscopic images showing the representative immunohistochemical staining against mKi67 in colorectal tumors from WT, *C1* KO, and *C2‐4* KO mice. L) Scatter plots represent the percentages of mKi67 positive staining signals per FOV in (K). Data are representative of at least three independent experiments (mean ± SEM in [A, C, E, F, H, J, L]). *p < 0.05, **p < 0.01, and ***p < 0.001 by Student's *t*‐test.

To explore the mechanism of GSDMC in promoting CRC development, we first established CT26 and MC38 mouse colorectal cancer cell lines stably overexpressing GSDMC2–4 or expressing shRNA targeting GSDMC2‐4 (shGSDMC) and confirmed the overexpression or knockdown of GSDMC2‐4 by qPCR and Western blot (Figure S6A,B, Supporting Information). Overexpression or knockdown of GSDMC2‐4 in CT26 and MC38 cells had no effect on cell proliferation in vitro, as assayed by Cell counting kit‐8 (CCK‐8) assay (**Figure** **3**A). However, overexpression of GSDMC2‐4 remarkably promoted tumor growth; while, knockdown of GSDMC2‐4 inhibited tumor growth in the subcutaneous tumor model for MC38 (Figure 3B,C) and CT26 (Figure 3D–F). To investigate whether GSDMC2, GSDMC3, and GSDMC4 played redundant roles in promoting tumor growth, we overexpressed GSDMC2, GSDMC3, and GSDMC4 individually in MC38 and CT26 cells and discovered that overexpression of any individual gene showed no apparent effect on tumor cell proliferation in vitro (Figure S6C,D, Supporting Information). Interestingly, overexpression of GSDMC2, GSMDC3, or GSDMC4 promoted tumor growth of MC38 cells in the subcutaneous tumor model (Figure S6E,F, Supporting Information), suggesting that any one of GSDMC2, GSMDC3, or GSDMC4 was sufficient to promote tumor growth in vivo. These data suggest that GSDMC2, GSDMC3, or GSDMC4 promote CRC development in a redundant manner. In addition, as the tumor microenvironment is one of the key factors driving the difference in tumor cell growth between in vitro and in vivo conditions, our data suggest that GSDMC2/3/4 might promote CRC progression by regulating the TME.

T cells in the TME play important roles in tumor progression. To determine whether GSDMC‐mediated tumor growth is dependent on T cells, nude mice were used to assess tumor growth in vivo. Our results showed that the effects of overexpression or knockdown of GSDMC2‐4 on MC38 tumor growth in nude mice were similar to those in C57BL/6 mice (Figure S6G, Supporting Information). Overexpression of GSDMC2‐4 in CT26 cells showed a trend but no significant difference in promoting tumor growth in nude mice; however, knockdown of GSDMC2‐4 inhibited tumor growth (Figure S6H, Supporting Information). These data suggest that GSDMC2/3/4‐mediated promotion of tumor growth in vivo is not solely dependent on T cells.

Overexpression of GSDMC significantly promoted in vivo tumor cell proliferation, as indicated by the increased Ki67 staining signal, as well as the increased intensity of microvessels (CD31 staining) in both CT26 tumors (Figure 3G,H) and MC38 tumors (Figure 3I,J). Further, in the AOM‐DSS induced CRC model, *Gsdmc2‐4* deficiency also led to decreased tumor proliferation, as shown by fewer Ki67^+^ cells in CRC tissues (Figure 3K,L). These results indicate that GSDMC2/3/4 promote tumor growth in vivo.

### 2.6 Hypoxia and Low‐Glucose Mediated GSDMC Activation Promotes Tumor Cell Pyroptosis to Facilitate Tumor Necrosis

**Figure 4:**
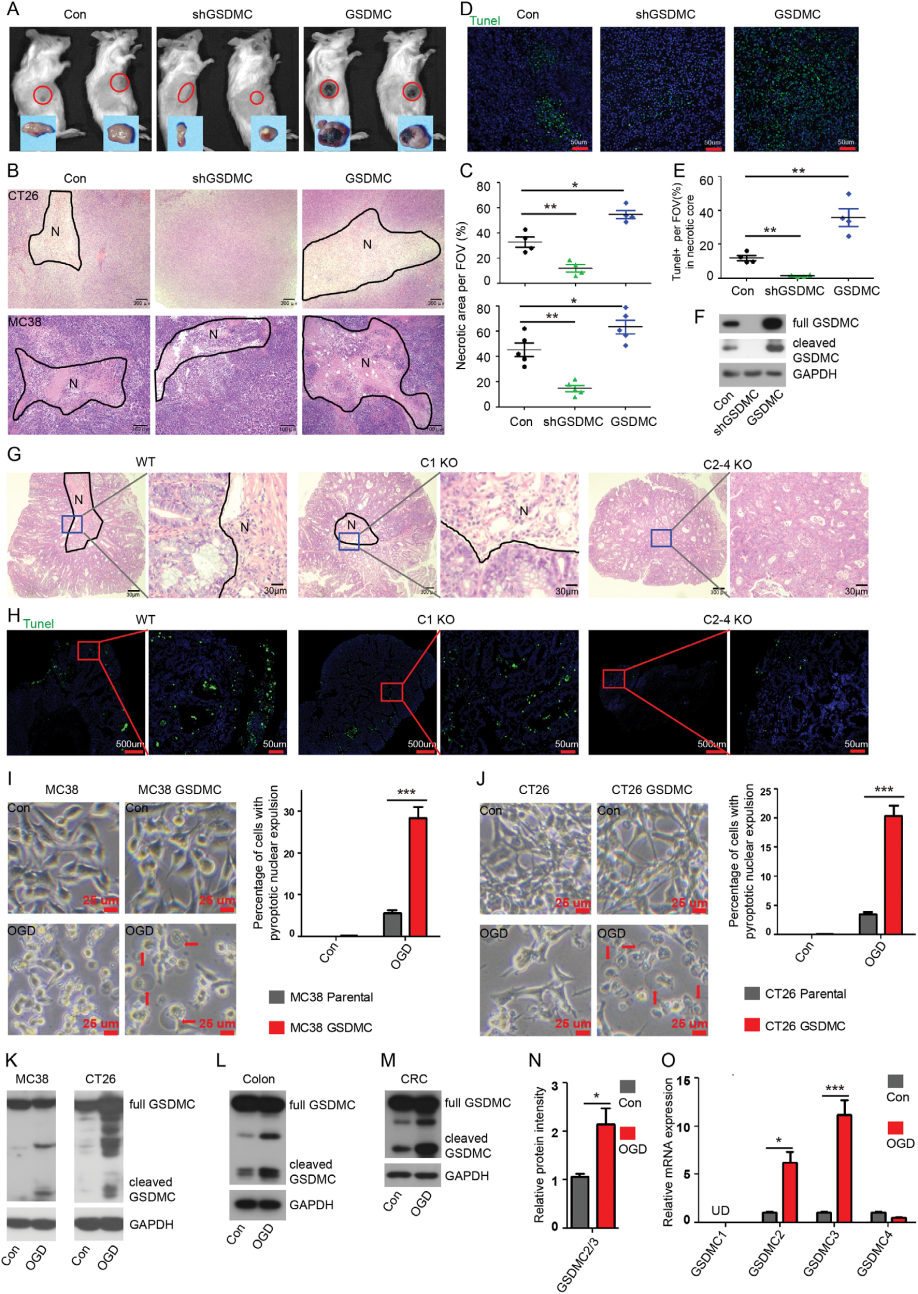
Hypoxia and low‐glucose stimuli activate GSDMC to mediate colorectal cancer cell pyroptosis and facilitate tumor necrosis. A) Macroscopic image showing the representative subcutaneous tumor grafts of CT26 cells stably expressing GSDMC shRNA, GSDMC2‐4, or control empty vectors. Red circles indicate the boundary of each subcutaneous tumor. The dissected tumor from each mouse is shown below. B,C) H&E histology of the representative CT26 (upper panel) and MC38 (lower panel) grafts. N indicates necrotic area (B). Scatter plots represent the percentages of necrotic area per FOV (C). D,E) Microscopic images showing the representative TUNEL staining in subcutaneous tumors (D). Scatter plots represent the percentages of positive TUNEL staining per FOV (E). F) Immunoblot of GSDMC in subcutaneous tumors from (A). G) H&E histology of the representative AOM‐DSS induced CRC from WT, *C1* KO, and *C2‐4* KO mice. N indicates necrotic area. H) Microscopic images showing the representative TUNEL staining in colorectal tumors as indicated in (G). Green is for positive TUNEL staining and blue is for nucleus. I,J) Representative images showing the morphology of MC38 cells (I) or CT26 cells (J) stably expressing GSDMC2‐4 or empty vectors under OGD treatment; the red arrows indicate pyroptotic nuclear expulsion with large bubbles. Bar graphs represent percentages of cells with nuclear expulsion bubbles in five randomly selected images. K–M) Immunoblot of GSDMC in MC38 and CT26 cells stably expressing GSDMC2‐4 (K), mouse colon (L), or mouse CRC (M) under OGD treatment. N) Quantitative analysis of GSDMC band intensity (a sum of full length GSDMC and cleaved fragments, normalized to GAPDH) in (M). O) Quantitative mRNA expression of GSDMC1/2/3/4 in mouse CRC tissues under OGD treatment. Data are representative of at least three independent experiments (mean ± SEM in [C, E, I, J, N, and O]). *p < 0.05, **p < 0.01, and ***p < 0.001 by Student's *t*‐test.

Hypoxia and low‐glucose conditions are commonly observed in regions surrounding necrotic tumor cells and could profoundly influence TME to limit immune responses.^[^ ^6^, ^23^, ^24^, ^25^ ^]^ Interestingly, we noticed that the GSDMC‐expressing CT26 tumors in syngeneic mice frequently displayed areas of necrosis compared to size‐matched control tumors (**Figure** **4**A). H&E staining revealed larger areas of necrosis in GSDMC‐expressing CT26 or MC38 tumors, while showing less necrosis in shGSDMC‐expressing tumors (Figure 4B,C). TUNEL staining showed elevated cell death in the necrotic core of GSDMC‐expressing CT26 tumors (Figure 4D,E). Increased GSDMC activation was observed in GSDMC‐expressing tumors, indicating enhanced cell pyroptosis, consistent with the TUNEL staining results (Figure 4F). Similarly, *Gsdmc2–4* deficiency dramatically decreased necrotic areas in CRC tissues (Figure 4G). Echoing the reduced necrosis, tumors from *Gsdmc2–4* deficient mice exhibited reduced TUNEL staining signals, indicating less cell death in *Gsdmc2–4* deficient tumors (Figure 4H). These data suggest that GSDMC2/3/4 might promote tumor cell pyroptosis, thereby facilitating tumor necrosis.

Given the increase in necrotic areas in GSDMC‐overexpressing tumors, we next sought to explore whether GSDMC could be activated by hypoxia and low‐glucose conditions to mediate cell pyroptosis. To this end, GSDMC‐overexpressing MC38 and CT26 cells, as well as the control cells, were cultured in oxygen‐glucose depleted (OGD) medium for 6 h. Pyroptotic nuclear expulsions were prominently observed in GSDMC‐overexpressing MC38 (Figure 4I) and CT26 (Figure 4J) cells under OGD treatment; while, control cells exhibited a significantly lower degree of such expulsions, suggesting that overexpression of GSDMC increased cell pyroptosis under OGD conditions. Further, OGD treatment resulted in the activation of GSDMC in MC38 and CT26 cells (Figure 4K), as well as in freshly isolated primary colon tissues and colorectal tumors from mice (Figure 4L,M). Statistical analysis of western blot band intensity showed increased expression of GSDMC2 and GSDMC3 at the protein level in colorectal tumors upon OGD treatment (Figure 4N), and the increased mRNA expression levels of GSDMC2 and GSDMC3 were also confirmed by qPCR (Figure 4O). These results suggest that hypoxia and low‐glucose conditions are sufficient to activate GSDMC in vitro and also induce GSDMC expression in colorectal tumor cells, indicating that physiological hypoxia and low‐glucose conditions might facilitate tumor necrosis through activating GSDMC.

### 2.7 Caspase‐6 is Responsible for Hypoxia and Low‐Glucose Induced GSDMC Activation in Colorectal Cancer

**Figure 5:**
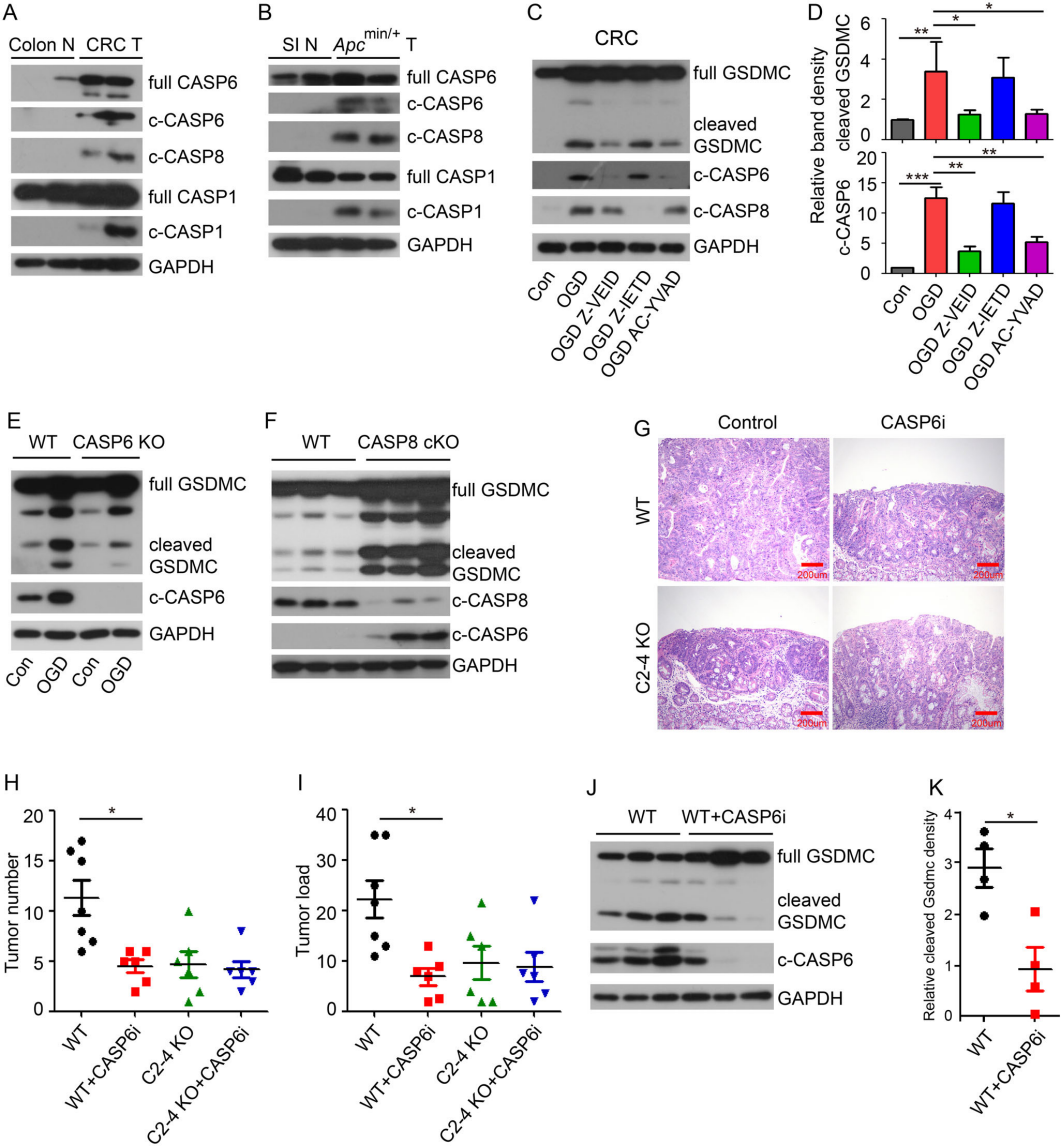
Caspase‐6 is responsible for hypoxia and low‐glucose induced GSDMC activation. A,B) Western blot of cleaved Caspase‐1 (c‐Casp1), c‐Casp6, and c‐Casp8 in mouse colon and CRC tissue (A), and intestinal tumors from *Apc^min/+^* mice and small intestine tissue (B). C,D) Western blot analysis of GSDMC, c‐Casp6, and c‐Casp8 in CRC under OGD treatment combined with or without Z‐VEID, Z‐IETD, and AC‐YVAD (C). Bar graphs represent quantitative analysis of cleaved N‐termial GSDMC and c‐Casp6, normalized to GAPDH band intensity (D). E) Western blot analysis of GSDMC and c‐Casp6 in colon tissues from wild‐type and Caspase‐6 knockout mice (*Casp6* KO). F) Western blot analysis of GSDMC, c‐Casp6, and c‐Casp8 in colon tissues from wild‐type and IEC‐specific Caspase‐8 knockout mice (*Casp8* cKO). G) H&E histology showing representative colorectal tumors from WT and *C2‐4* KO mice in AOM‐DSS induced CRC model with or without administration of Caspase‐6 inhibitor Z‐VEID (CASP6i). H,I) Tumor number (H) and tumor load (I) in the colons as indicated in (G). J) Western blot analysis of GSDMC and c‐Casp‐6 in CRC. K) Quantitative analysis of cleaved N‐terminal GSDMC band intensity in (J). Colon N, Normal colon; CRC T, CRC tissue; SI N, Normal small intestine.

GSDMC was previously shown to be cleaved by Caspase‐6 and Caspase‐8 in vitro and was activated by Caspase‐8 but not by Caspase‐6 in breast cancer.^[^ ^16^ ^]^ To investigate which Caspase protein might be responsible for GSDMC activation in colorectal cancer, we first analyzed the expression of various Caspase proteins in intestinal tumors. We found that Caspase‐1, Caspase‐6, and Caspase‐8 were expressed and activated in mouse CRC and small intestinal tumors (**Figure** **5**A,B). In addition, analysis of the human TCGA dataset for CRC revealed that the median mRNA expression levels of Caspase‐6 and Caspase‐8 were significantly higher in tumors; while, Caspase‐1 expression remained unchanged (Figure S7A–C, Supporting Information). As GSDMC activation could be induced by OGD treatment (Figure 4H–J), we next sought to determine which Caspase was responsible for GSDMC activation under these conditions. Using an ex vivo culture system with OGD treatment, we observed that both Caspase‐6 and Caspase‐8 were activated in mouse CRC tissues (Figure 5C). To identify which Caspase mediated GSDMC activation, we pretreated CRC tissues with the Caspase‐1 inhibitor AC‐YVAD, the Caspase‐6 inhibitor Z‐VEID or the Caspase‐8 inhibitor Z‐IETD, followed by OGD treatment. We found that the Caspase‐6 inhibitor Z‐VEID inhibited both Caspase‐6 activity and GSDMC activation; while, the Caspase‐8 inhibitor Z‐IETD inhibited Caspase‐8 activity but not GSDMC activation (Figure 5C,D). The Caspase‐1 inhibitor AC‐YVAD also inhibited both Caspase‐6 activity and GSDMC activation (Figure 5C,D), consistent with previous reports that Caspase‐6 can be activated by Caspase‐1.^[^ ^26^, ^27^, ^28^ ^]^ To further confirm the role of Caspase‐6 in activating GSDMC in mouse colon and CRC tissues, colons isolated from Caspase‐6 knockout (*Casp6* KO) mice were cultured ex vivo with OGD treatment, and GSDMC activation was significantly reduced in *Casp6* KO colon tissues (Figure 5E). It is worth noting that GSDMC activation was not completely inhibited in *Casp6* KO colons, indicating that additional mechanisms of GSDMC activation exist beyond Caspase‐6. Although the cleavage of GSDMC by Caspase‐8 was reported in breast cancer,^[^ ^16^ ^]^ conditional deletion of Caspase‐8 in intestinal epithelial cells dramatically enhanced GSDMC activation rather than inhibiting it, likely due to increased Caspase‐6 activity (Figure 5F). Thus, our data demonstrate that Caspase‐6, but not Caspase‐8, activates GSDMC in intestinal cancer.

To further investigate the effect of Caspase‐6‐mediated GSDMC activation on tumor development, we intraperitoneally injected vehicle or 5mg kg^−1^ Caspase‐6 inhibitor Z‐VEID‐FMK every other day for 30 days, starting at day 40 of the AOM‐DSS induced CRC mouse model. Interestingly, administration of Z‐VEID‐FMK significantly suppressed CRC tumor progression in WT mice (Figure 5G–I). Both tumor number and tumor load in Z‐VEID‐FMK‐treated WT mice were reduced to levels comparable to those in *Gsdmc2–4* deficient mice; while, Z‐VEID‐FMK had no further suppressive effect on tumor development in *Gsdmc2–4* deficient mice (Figure 5H,I). To verify whether inhibition of Caspase‐6 by Z‐VEID‐FMK in vivo affects GSDMC activation, we demonstrated that Z‐VEID‐FMK inhibited Caspase‐6 activity, and subsequently, GSDMC activation (Figure 5J,K). Taken together, our data indicate that Caspase‐6 activates intestinal epithelial GSDMC to promote colorectal tumor progression.

### 2.8 Tumor Cell‐Derived CXCL2 Recruits MDSCs into Tumor Microenvironment

To elucidate the mechanism of GSDMC‐mediated CRC development, we performed transcriptomic analysis of AOM‐DSS induced CRC tissues from WT control, *Gsdmc1* ^−/−^, and *Gsdmc2‐4* ^−/−^ mice, and identified 892 differentially expressed genes (Figure S8A, Supporting Information). By comparing the transcriptomic profiles of CRC tissues from WT and *Gsdmc2–4* KO mice, bioinformatic analysis revealed enrichment of humoral immune response, cellular response to LPS, and innate immune response in mucosa as the top upregulated pathway signatures; while, epithelial cell differentiation, regulation of leukocyte tethering, and regulation of pH were identified as downregulated pathway signatures (Figure S8B, Supporting Information). These findings suggest that the inflammatory state in tumors might account for the major differences observed.

**Figure 6:**
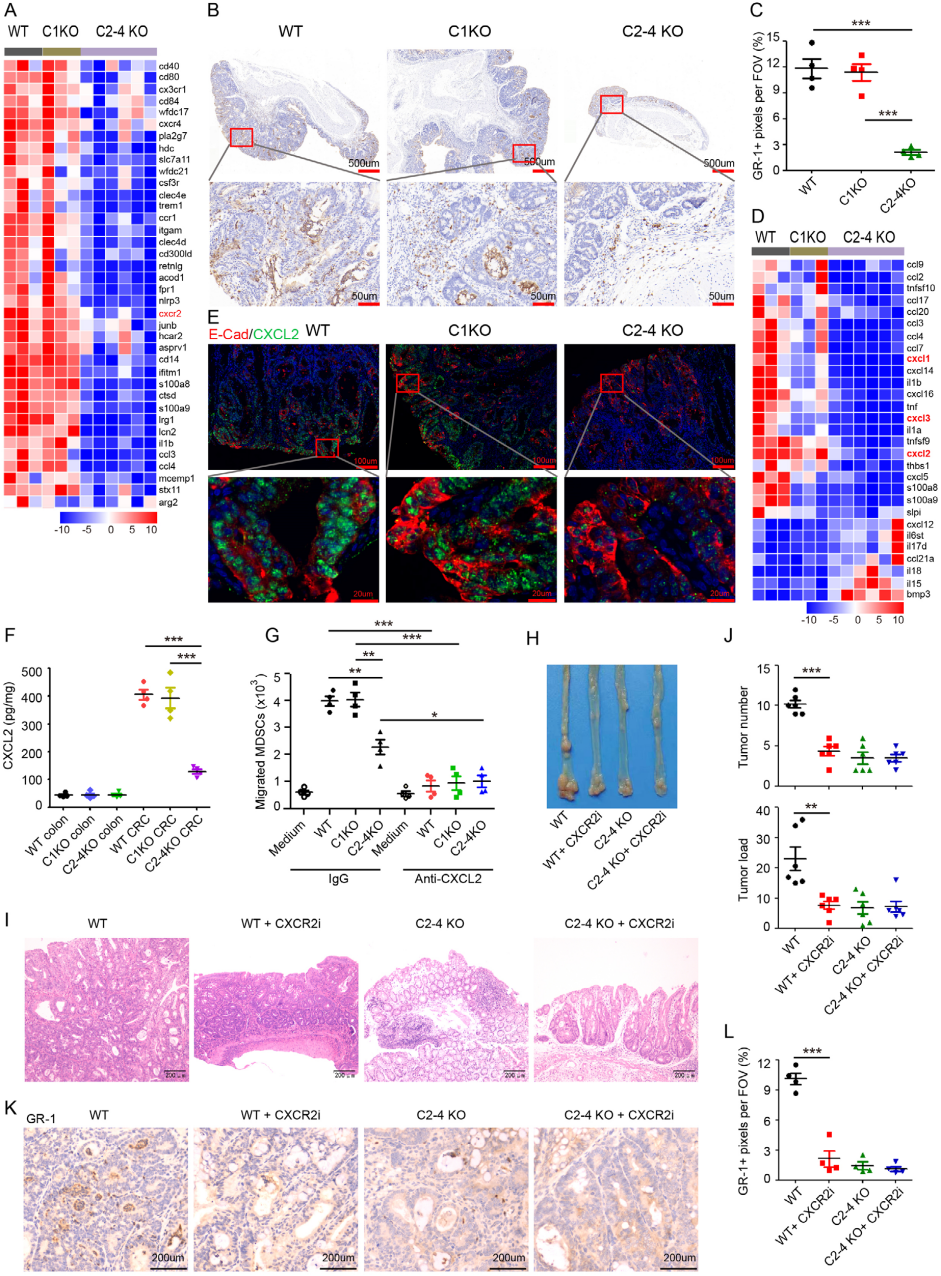
GSDMC2/3/4 promote tumor cell expression of CXCL2, which recruits MDSCs into the tumor microenvironment. A) Heatmap depicting differentially expressed genes associated with MDSCs from transcriptome sequencing data. B) Microscopic images showing the representative immunohistochemical staining against GR‐1 in CRC from WT, *C1* KO, and *C2‐4* KO mice. C) Scatter plots represent the percentages of positive GR‐1 staining signals per FOV. D) Heatmap depicting differentially expressed cytokines/chemokines from transcriptome sequencing data. E) Immunofluorescence staining of E‐cad (Red), CXCL2 (Green) in CRC. F) The protein level of CXCL2 in CRC lysate was measured with ELISA kits. G) Migration of MDSCs toward conditioned medium (CM) in the presence of IgG control or CXCL2‐neutralizing antibodies. Migrated cells across transwell membrane were counted. H,I) Macroscopic view (H) and H&E histology (I) of the representative colons from WT and *C2‐4* KO mice of the AOM‐DSS model with or without administration of CXCR2 inhibitor. J) Tumor number and tumor load in the colons from mice as indicated in (H). K) Microscopic images showing the representative immunohistochemical staining against GR‐1 in CRC as indicated in (H). L) Scatter plots represent the percentages of positive GR‐1 staining signals per FOV. Data are representative of at least three independent experiments (mean ± SEM in [C, F, G, J, and L]). *p < 0.05, **p < 0.01, and ***p < 0.001 by Student's *t*‐test.

The results described above indicated that GSDMC might promote intestinal tumor development by regulating the TME. Therefore, we focused on genes that might regulate the TME. We identified a set of downregulated genes associated with the functionality of MDSCs^[^ ^29^, ^30^, ^31^, ^32^ ^]^ in *Gsdmc2–4* ^−/−^ CRC (**Figure** **6**A). MDSCs are a functional myeloid cell subset with immunosuppressive properties in inflammation, particularly prominent in late‐stage cancers. In mice, MDSCs are commonly defined by the cell surface markers CD11b and GR‐1.^[^ ^33^, ^34^, ^35^, ^36^ ^]^ Immunohistochemical staining of CRC tissues revealed decreased infiltration of MDSC (positive for GR‐1) in *Gsdmc2–4* KO CRC (Figure 6B,C). Flow cytometry analysis further confirmed a significantly decreased percentage of MDSCs (defined as CD45^+^ CD11b^+^ F4/80^−^ GR‐1^+^)^[^ ^33^ ^]^ in *C2–4* KO CRC tissues (Figure S8C,D, Supporting Information). Specifically, polymorphonuclear MDSCs (PMN‐MDSCs), defined as CD45^+^ CD11b^+^ F4/80^−^ GR‐1^+^ Ly6G^+^, dominated the MDSC population in WT and *C1* KO CRC tissues; while, their percentages were significantly reduced in *C2–4* KO CRC tissues (Figure S8C,D, Supporting Information). These data indicate that GSDMC2/3/4 promote the recruitment of MDSCs (primarily PMN‐MDSCs) during CRC development.

Several chemoattractants, such as CXCL1, CXCL2, CXCL3, CXCL5, CXCL7, and CXCL15, have been reported to recruit MDSCs through binding to CXCR2.^[^ ^37^ ^]^ Transcriptomic analysis revealed a significant downregulation of CXCL1, CXCL2, CXCL3, and CXCL5 expression in *C2–4* KO CRC tissues compared to WT and *C1* KO CRC tissues (Figure 6D). QPCR analysis demonstrated that the expression levels of CXCL1, CXCL2, and CXCL3 were significantly upregulated in CRC tissues compared to normal colon tissues. Further, a marked downregulation of CXCL1, CXCL2, and CXCL3 expression was observed in *C2–4* KO CRC tissues relative to WT CRC tissues, whereas the expression levels of CXCL5, CXCL7, and CXCL15 remained unchanged between *C2–4* KO and WT CRC tissues (Figure S9A, Supporting Information). Next, we confirmed the expression of these CXCLs by immunofluorescence staining and found that CXCL2 was highly expressed in E‐cad^+^ tumor cells (Figure 6E). Importantly, *Gsdmc2–4* deficiency dramatically decreased CXCL2 expression in CRC tissues (Figure 6E). Unlike CXCL2, CXCL1 was primarily expressed in α‐SAM^+^ fibroblast (Figure S9B, Supporting Information). ELISA assay showed that CXCL1 protein levels were elevated in CRC compared to normal colon tissues; however, no significant difference was observed among WT, *Gsdmc1* deficient, and *Gsdmc2–4* deficient tumors (Figure S9C, Supporting Information). Consistent with the transcriptomic data and immunofluorescence staining, CXCL2 protein levels were further confirmed to be dramatically elevated in CRC tissues, and *Gsdmc2–4* deficiency attenuated this upregulation (Figure 6F). Although the mRNA expression of CXCL3 was increased in CRC tissues, it was undetectable in mouse CRC by immunofluorescence staining (data not shown), possibly due to its relatively low expression abundance. These findings demonstrate that CXCL2 expression is increased in colorectal cancer, and *Gsdmc2–4* deficiency suppresses the induction of CXCL2 expression in E‐cad^+^ tumor cells.

To investigate whether GSDMC2/3/4 promote MDSC recruitment through CXCL2, we performed transwell assays. Conditioned media from CRC tissues induced the migration of MDSCs, and the number of migrated MDSCs was decreased in conditioned media from *Gsdmc2–4* deficient CRC tissues (Figure 6G). Interestingly, the migration of MDSCs was nearly abolished by the addition of an anti‐CXCL2 neutralizing antibody (Figure 6G). Thus, our data indicate that GSDMC2/3/4 promote the expression of CXCL2, which facilitates the recruitment of MDSCs into the tumor microenvironment.

### 2.9 GSDMC2/3/4 Promote Colorectal Cancer Development Through CXCL2‐CXCR2 Axis‐Dependent MDSC Recruitment

CXCR2 is the receptor for CXCLs, and deletion of CXCR2 has been shown to inhibit the infiltration of MDSCs into the colonic mucosa and tumors.^[^ ^38^ ^]^ Indeed, MDSCs in CRC tissues highly expressed CXCR2, and immunofluorescence staining revealed co‐localization of CXCR2 with GR‐1 (Figure S9D, Supporting Information). To determine whether decreased MDSC infiltration was responsible for the attenuated CRC progression in *Gsdmc2–4* deficiency mice, we intraperitoneally injected vehicle or 10 mg kg^−1^ CXCR2 antagonist SB225002 every 3 days for 30 days, starting at day 40 of the AOM‐DSS induced CRC model. Treatment with CXCR2 antagonist attenuated CRC development in WT mice but had no effect on CRC development in *C2–4* KO mice (Figure 6H–J). Further, CXCR2 antagonist treatment significantly reduced MDSC infiltration in CRC tissues (Figure 6K,L), which was accompanied by enhanced T cell infiltration (Figure S9E, Supporting Information). These findings are consistent with the established role of MDSCs in suppressing T cell‐mediated anti‐tumor immunity.^[^ ^33^ ^]^ Comparative analysis revealed that WT CRC tissues exhibited significantly higher levels of angiogenesis, as evidenced by CD31 immunostaining, than *C2–4* KO CRC tissues (Figure S9G, Supporting Information). To investigate the potential correlation between MDSC infiltration and angiogenesis, we evaluated the effects of CXCR2 antagonist SB225002 treatment on angiogenesis in vivo. The results demonstrated that SB225002 treatment markedly suppressed angiogenesis in WT CRC tissues, while, having no significant effect on *C2–4* KO CRC tissues. These findings suggest that the enhanced angiogenesis observed in WT CRC tissues is mediated through MDSC‐dependent mechanisms, which aligns with previous reports.^[^ ^35^ ^]^ These data demonstrate that GSDMC2/3/4 promote colorectal tumor development through CXCL2/CXCR2‐mediated recruitment of MDSCs into the tumor microenvironment.

### 2.10 GSDMC2/3/4‐Mediated HMGB1 Release from Pyroptotic Tumor Cells Induces Cxcl2 Expression in Colorectal Cancer Cells

**Figure 7:**
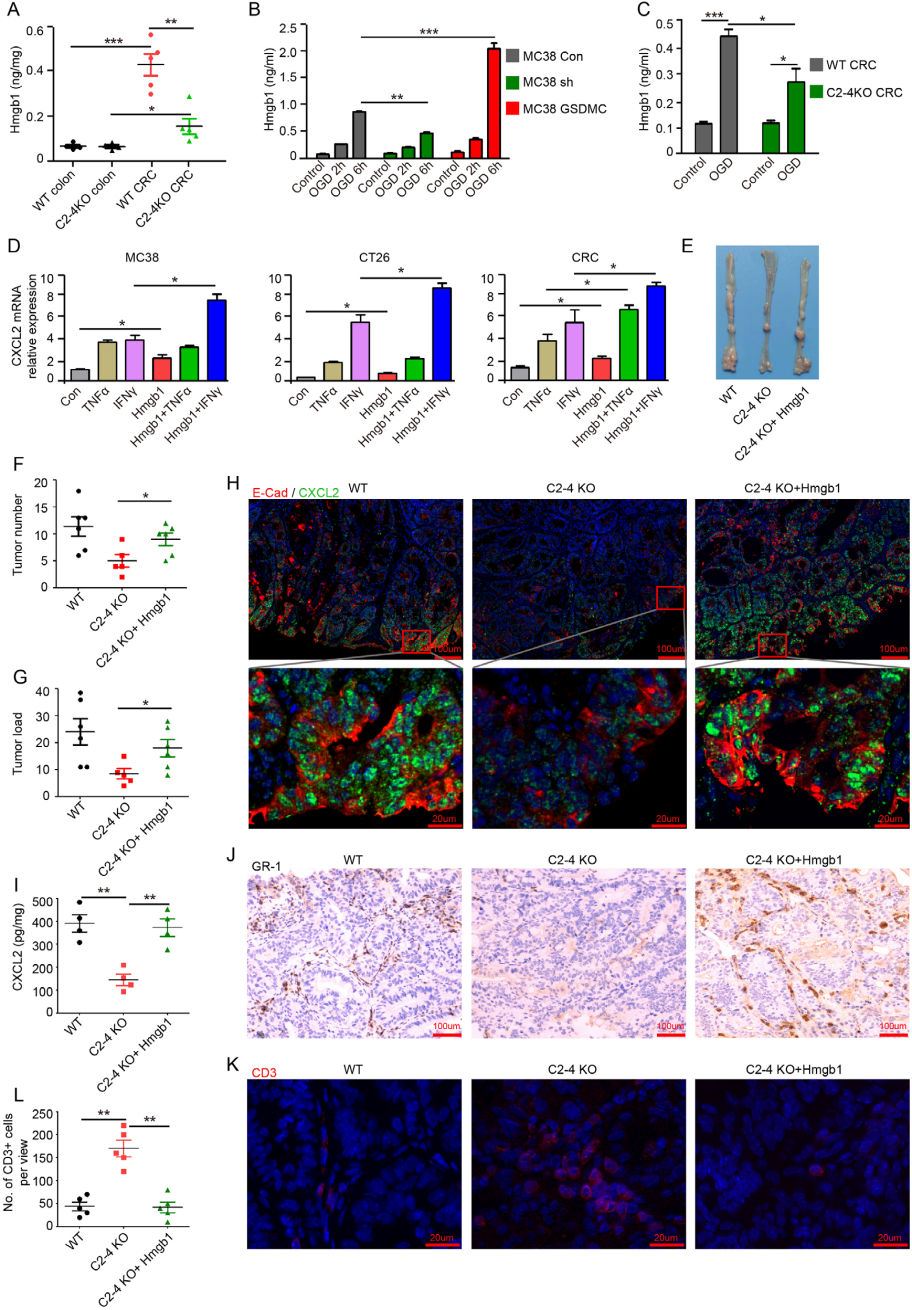
GSDMC2/3/4 promote HMGB1 release, which enhances CXCL2 expression in colorectal cancer. A) The protein levels of HMGB1 in interstitial fluid of colon tissues or CRC were measured with ELISA kit. B,C) HMGB1 release in culture supernatant of MC38 cells (B) or CRC tissues (C) under OGD treatment was measured with ELISA kit. D) Quantitative mRNA expression of CXCL2 in MC38, CT26, and CRC tissues cultured with 10 ng mL^−1^ TNFα, 20 ng mL^−1^ IFNγ, or 1 µg mL^−1^ HMGB1, or HMGB1 plus TNFα or IFNγ for 24 h. E) Macroscopic view of the representative colons from WT and *C2‐4* KO mice of AOM‐DSS induced CRC model with or without administration of HMGB1. F,G) Tumor number (F) and tumor load (G) from mice as indicated in (E). H) Immunofluorescence staining of E‐cad and CXCL2 in CRC as indicated in (E). I) The protein level of CXCL2 in CRC lysate was measured with ELISA kit. J) Representative immunohistochemical staining against GR‐1 in CRC as indicated in (E). K) Immunofluorescence staining of CD3 in CRC as indicated in (E). L) Number of CD3^+^ cells was counted in immunofluorescence staining slides (K) in 20× magnification fields. Data are representative of at least two independent experiments (mean ± SEM in [A–D, F, G, I, L]). *p < 0.05, **p < 0.01, and ***p < 0.001 by Student's *t*‐test.

GSDMs‐mediated cell pyroptosis typically releases intracellular contents including immunogenic damage‐associated molecular patterns (DAMPs), such as HMGB1, lactoferrin (LTF), S100 proteins A8 and A9 (S100A8/9), IL1α, IL33, fibrinogen, and fibronectin.^[^ ^39^ ^]^ An analysis of DAMP expression patterns in various cell types from colon tissue identified HMGB1 as primarily expressed in intestinal epithelial cells, and S100A8/9 as primarily expressed in neutrophils (Figure S10, Supporting Information). HMGB1 is known to function as an immunomodulatory cytokine. We next investigated whether HMGB1 was involved in GSDMC‐mediated intestinal tumor progression. Indeed, we observed elevated HMGB1 release in CRC interstitial fluid (**Figure** **7**A). Interestingly, *Gsdmc2–4* deficiency decreased the release of HMGB1 from CRC tissues (Figure 7A). Overexpression of GSDMC drastically augmented HMGB1 release; while, knockdown of GSDMC prevented its release from MC38 cells under OGD treatment (Figure 7B). Similarly, OGD treatment promoted HMGB1 release from WT CRC tissues; while, loss of *Gsdmc2–4* suppressed its release (Figure 7C). We next examined whether HMGB1 was responsible for the increased CXCL2 expression in CRC. HMGB1 alone induced the expression of CXCL2 in MC38, CT26, and CRC tissues (Figure 7D). Although TNFα and IFN‐γ have long been implicated in antitumor immune responses, recent reports suggested that they may also play pro‐tumorigenic roles through the development of an immunosuppressive TME.^[^ ^40^, ^41^ ^]^ We found that HMGB1, in combination with IFN‐γ, synergistically promoted CXCL2 expression in MC38, CT26, and CRC tissues (Figure 7D), and HMGB1 in combination with TNFα also synergistically increased CXCL2 expression in CRC tissues (Figure 7D). These results highlight the role of HMGB1, released from pyroptotic tumor cells, in upregulating CXCL2 expression.

To examine whether HMGB1 could restore CXCL2 expression and CRC tumorigenesis in *Gsdmc2–4* deficient mice, we intraperitoneally injected vehicle or 2 µg per mouse recombinant HMGB1 every other day for 30 days in the AOM‐DSS model. Recombinant HMGB1 obviously restored tumor formation, tumor number, and tumor load in *Gsdmc2–4* ^−/−^ mice (Figure 7E–G). Consistently, in vivo HMGB1 administration significantly increased CXCL2 protein expression in *Gsdmc2–4* deficient CRC (Figure 7H,I). Moreover, in accordance with the recovery of CXCL2 expression, infiltration of MDSCs was correspondingly increased in *Gsdmc2–4* deficient CRC (Figure 7J). Further, HMGB1 treatment significantly decreased CD3^+^ T cell infiltration in *Gsdmc2–4* deficient CRC (Figure 7K,L). These results indicate that HMGB1, released from GSDMC‐mediated pyroptotic cells, enhances CXCL2 expression in tumor cells, and the elevated CXCL2 subsequently recruits CXCR2^+^ MDSCs to orchestrate the immunosuppressive TME.

## 3 Discussion

**Figure 8:**
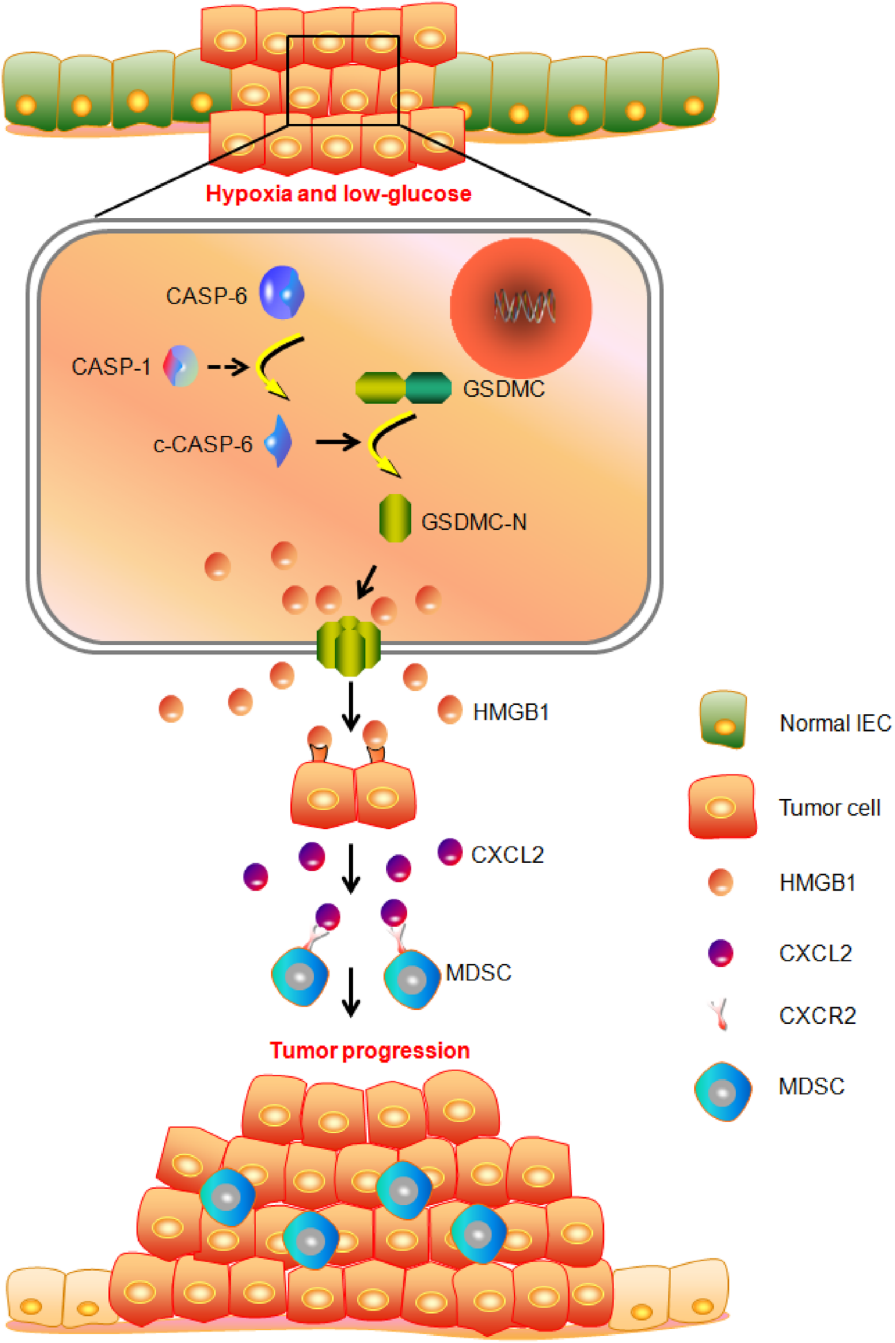
The schematic diagram shows that Caspase‐6/Gasdermin C‐mediated tumor cell pyroptosis promotes colorectal cancer progression through CXCL2‐dependent recruitment of myeloid‐derived suppressor cells.

Hypoxia and low‐glucose condition is a well‐known feature of solid tumors.^[^ ^6^, ^7^ ^]^ However, the molecular mechanisms of Hypoxia and low‐glucose condition in regulating intestinal cancer development remain largely elusive. Here, we found that Hypoxia and low‐glucose conditions lead to colorectal tumor cell pyroptosis through Caspase‐1/Caspase‐6/GSDMC2/3/4 axis in mouse model. The enhanced release of HMGB1 from pyroptotic tumor cells subsequently increases the expression of CXCL2 in neighboring tumor cells, which recruit MDSCs into the tumor microenvironment, thereby promoting CRC development (**Figure** **8**).

In this study, we identified that the mRNA expression levels of GSDMC2/3/4 were high in colon, whereas the expression of GSDMC1 was relatively low, which was consistent with a previous report.^[^ ^42^ ^]^ Therefore, we constructed *Gsdmc2–4* triple deficiency mice and *Gsdmc1* deficiency mice and revealed that deficiency of *Gsdmc2–4* suppressed colorectal cancer development. Further, we found that overexpression of GSDMC2, GSDMC3, or GSDMC4 in MC38 cells individually was sufficient to promote tumor growth in vivo. Our study first demonstrated that GSDMC2/3/4 but not GSDMC1 were essential for CRC development, and GSDMC2, GSDMC3, or GSDMC4 might play a redundant role in promoting tumor growth. To verify this finding, deficiency of GSDMC2, GSDMC3, and GSDMC4 individually in tumor cells or mice should be used to assess the role of GSDMC2, GSDMC3, and GSDMC4 in CRC development in the future.

We found that OGD treatment increased GSDMC expression in CRC tissues, suggesting that increased GSDMC expression in CRC might be regulated by hypoxia and low‐glucose condition. Hou et al. found that nuclear PD‐L1 formed a complex with p‐Stat3 to increase the transcription of the GSDMC gene under hypoxia in breast cancer cell line.^[^ ^16^ ^]^ Whether OGD conditions induce GSDMC expression in CRC through similar mechanism needs to be investigated in the future.

Hou et al. found that under hypoxia conditions, TNFα plus CHX induced breast cancer cell pyroptosis through Caspase‐8 mediated GSDMC cleavage.^[^ ^16^ ^]^ In the present study, we found that hypoxia and low‐glucose directly induced colon cancer cell pyroptosis through Caspase‐6 mediated GSDMC activation. We surprisingly found that conditional deletion of Caspase‐8 in intestinal epithelial cells did not suppress but increased GSDMC activation, and the increased GSDMC activation might account for enhanced Caspase‐6 activity. We found that Caspase‐6, but not Caspase‐8, mediated OGD‐induced GSDMC activation in colon cancer cells. Using Caspase specific inhibitors and Caspase‐6 knockout mice, we further demonstrated that Caspase‐6 was required for GSDMC activation in colon and CRC tissues under Hypoxia and low‐glucose conditions. Interestingly, Caspase‐6 deficiency dramatically reduced but did not completely block OGD‐induced GSDMC activation, suggesting other regulators might function to activate GSDMC in intestine in the absence of Caspase‐6. Our results also indicated that Caspase‐1 might act as an upstream regulator for Caspase‐6 activation, and subsequently, for GSDMC activation. Thus, our data revealed a new mechanism of GSDMC activation by Caspase‐6 to induce colon cancer cell pyroptosis under Hypoxia and low‐glucose conditions, which was different from Caspase‐8 mediated GSDMC activation in breast cancer. In addition, administration of Caspase‐6 inhibitor in vivo suppressed GSDMC activation and decreased CRC development fairly to the levels of *Gsdmc2–4* deficient mice. Our data suggested that Caspase‐6 activity was required for GSDMC activation in colon cancer both in vitro and in vivo.

The immature myeloid cells accumulate in the tumor microenvironment (TME) and acquire their maximal immunosuppressive functions after a phase of activation. MDSC recruitment to the TME is a crucial step for the development of an immunosuppressive milieu and is mainly mediated by the chemokine receptor CXCR2 which is expressed on PMN‐MDSCs. Thus, PMN‐MDSCs are recruited primarily by CXC chemokines, including CXCL1, CXCL2, and CXCL5.^[^ ^43^ ^]^ In the present study, HMGB1 released from pyroptotic tumor cells induced the upregulation of CXCL2 in tumor cells; thus, contributing to the fundamental recruitment of PMN‐MDSCs. The development of MDSCs exacerbated the existing imbalance in inflammation‐associated lesions, thereby contributing to the tumor development. The immunosuppressive effects of MDSCs are primarily mediated through the production of inhibitory molecules such as arginase‐1, inducible nitric oxide synthase (iNOS), and reactive oxygen species (ROS), as well as the secretion of immunosuppressive cytokines including IL‐10 and TGF‐β, which collectively inhibit the anti‐tumor functions of T cells and natural killer cells. MDSCs can also contribute to tumor growth via non‐immunological functions such as establishing and sustaining the tumor vasculature.^[^ ^35^ ^]^ Therefore, MDSCs might facilitate GSDMC‐mediated CRC progression by immune modulation and enhancement of angiogenesis.

In conclusion, we have identified *Gsdmc2/3/4* as critical oncogenes that promote CRC development. Our results demonstrate the upregulation and activation of GSDMC2/3/4 by Caspase‐6 in colorectal cancer cells under Hypoxia and low‐glucose conditions. Activation of GSDMC2/3/4 induces tumor cell pyroptosis and facilitates the release of HMGB1, which enhances the expression of CXCL2 in tumor cells. Our study provides a mechanism by which GSDMC2/3/4‐mediated cell pyroptosis, in response to Hypoxia and low‐glucose, promotes CRC progression through CXCL2‐dependent MDSCs recruitment. These data suggest that GSDMC2/3/4 are potentially promising new targets for CRC therapy.

## 4 Experimental Section

### Reagents

Recombinant mouse TNFα (RP01702), mouse IFNγ (RP01070), and mouse HMGB1 (RP01545) were purchased from Abclonal Technology. Z‐VEID‐FMK (A1923), Z‐IETD‐FMK (B3232), and Z‐YVAD‐FMK (A8955) were purchased from APExbio.

CXCL1 ELISA Kit (RK00038), CXCL2 ELISA Kit (RK00165), and HMGB1 ELISA Kit (RK06737) were purchased from Abclonal Technology. RIPA lysis buffer (P0013C) was obtained from Beyotime Biotechnology. Azoxymethane (AOM, A5486) was from Sigma–Aldrich. Dextran sulfate sodium salt (DSS, MB5535) was from Meilunbio. Trizol reagent (15596026CN) was from Invitrogen. PrimeScript RT Master Mix (RR036A) and TB Green Premix *Ex Taq* (Tli RNaseH Plus) (RR420) were from Takara Bio. Protease inhibitor cocktail (P8340) was purchased from MERCK. Mouse Myeloid‐Derived Suppressor Cell Isolation Kit (130‐094‐538) was from Miltenyi Biotec. DMEM (G4511) and DMEM low glucose (G4520) were from Wuhan Servicebio Technology.

Anti‐mouse Gsdmc2/3 (ab229896) was from Abcam. Anti‐human Gsdmc (30469‐1‐AP) was from Proteintech. Anti‐cleaved Caspase‐6 (9761s) and anti‐cleaved Caspase‐8 (8592s) were from Cell Signaling Technology. Anti‐Caspase‐1 (AG‐20B‐0042‐C100) was from Adipogen. Anti‐Cxcl1 (12335‐1‐AP) and anti‐Cxcl2 (26791‐1‐AP) were from Proteintech Group. Anti‐GR‐1 (108402) was from Biolegend. Anti‐GSDMC (A14550, for mouse GSDMC4), Anti‐α‐Smooth Muscle Actin (ACTA2) Rabbit mAb (A17910), and Anti‐Caspase‐6 (A19559) were purchased from Abclonal. Anti‐E‐cadherin (GB12083), anti‐CD31 (GB12063), anti‐Ki67 (GB151499), and anti‐CD3 (GB13014) were from Wuhan Servicebio Technology. Anti‐mouse F4/80 (12‐4801‐82) was from Invitrogen. Anti‐mouse CD45 (147706), Anti‐mouse CD11b (101212), Anti‐mouse GR‐1 (108405), and Anti‐mouse Ly6G (127618) were from Biolegend.

### Cell Culture

MC38 and CT26 cells were maintained with DMEM containing 10% v/v FBS, penicillin (100 U mL^−1^), and streptomycin (100 µg mL^−1^) at 37 °C in a 5% CO_2_ incubator. For oxygen and glucose deprivation treatment, cells were cultured in a hypoxia chamber (92% N_2_, 5% CO_2_, and 3% O_2_) with DMEM low glucose containing 10% FBS which was pre‐incubated in a hypoxia chamber for 12 h.

### Plasmids Construction

The coding sequences of mice GSDMC2, GSDMC3, and GSDMC4 were synthesized and cloned into vector pLVX‐EF1a‐IRES‐Puro.

shGSDMC:CCGGGCATCTTACAGCCAAACTTCTCGAGAAGTTTGGCTGTAAGATGCTTTTTGAATT, which targets mouse GSDMC, and shControl: CCGGGCGCGCTTTGTAGGATTCGCTCGAGCGAATCCTACAAAGCGCGCTTTTTGAATT, were synthesized and cloned into vector pLKO.1‐U6‐EF1a‐copGFP‐T2A‐puro.

### Human Tissue Microarray

Tissue microarray of paired human colorectal tumors and adjacent non‐tumor tissues was purchased from Shanghai Outdo Biotech Co., Ltd., China. It contained paired tissues from 38 male patients and 37 female patients. Among them, 53 tumors were ranked as WHO grade 1/2 and 22 tumors, grade 3. 1 mm cores were cut from the corresponding paraffin‐embedded blocks, and 5 µm sections were used for immunohistochemical staining. Immunohistochemical scoring for GSDMC was evaluated as product of staining intensity (0, 0.5, 1, 2, 3) and positive rate (0–100%) in each 1 mm tissue core. A core was excluded if it contained poor nuclei staining. A total of 72 colorectal tumors and 67 para‐carcinoma normal tissues were included in the analyses.

### Mice

*Gsdmc1* ^−/−^ (*C1* KO) mice, *Gsdmc2–4* ^−/−^ (*C2‐4* KO) mice, *Apc* ^min/+^ mice, and *Caspase‐6* ^−/−^ (*Casp6* KO) mice on the C57BL/6 background were purchased from GemPharmatech (Nanjing, China). *Apc* ^min/+^ mice on the C57BL/6 background were crossed to *Gsdmc1* ^−/−^ mice and *Gsdmc2–4* ^−/−^ mice to generate *Apc* ^min/+^ *Gsdmc1* ^−/−^ mice and *Apc* ^min/+^ *Gsdmc2–4* ^−/−^ mice. Wild type C57BL/6, BALB/c, and BALB/c nude mice were purchased from Guangdong Medical Laboratory Animal Center. All mice were maintained in specific pathogen‐free conditions. All animal experiments were performed in compliance with the guidelines for the care and use of laboratory animals and were approved by the institutional biomedical research ethics committee of Guangdong Medical University.

### Induction of Colitis

6–8‐week‐old wild type and *Gsdmc1* ^−/−^, *Gsdmc2–4* ^−/−^ mice were given with 3% DSS in drinking water for 5 days, followed by normal drinking water until day 9. Mice were sacrificed for tissue analyses on day 9. For survival analysis, mice were given 3.5% DSS in drinking water for 5 days, followed by normal drinking water until day 13.

### AOM Plus DSS Induced Colorectal Cancer

Induction of colitis associated colorectal cancer was performed as previously described.^[^ ^44^ ^]^ Briefly, *Gsdmc1* ^−/−^, *Gsdmc2–4* ^−/−^, and their wild type littermates were injected with 10 mg kg^−1^ body weight AOM intraperitoneally. After 5 days, mice were given 2.5% DSS in drinking water for 5 days, followed by regular water for 16 days. Then, this cycle was repeated by 2.5% DSS for 5 days and with regular water for 16 days. Mice were given 2.0% DSS for 4 days in the third cycle and sacrificed for analyses on day 75 of the experiments. Macroscopic colon tumors were counted and measured with the caliper. Tumor load was calculated as the sum of all tumors’ diameters presented in a given mouse.

### Spontaneous Intestinal Cancer

Sex and age‐matched *Apc* ^min/+^ *Gsdmc1* ^−/−^, *Apc* ^min/+^ *Gsdmc2–4* ^−/−^, and *Apc* ^min/+^ littermates were allowed to develop intestinal tumors spontaneously for 20 weeks, and then, these mice were sacrificed for analyses.

### Bone Marrow Chimeras

To validate the effects of genetic deficiency of GSDMC when confined to either circulating cells (*Gsdmc2–4* ^−/−^ → WT) or nonhematopoietic tissue (WT → *Gsdmc2–4* ^−/−^), bone marrow transfer was performed to create *Gsdmc2–4* ^−/−^ chimera mice. Briefly, 8‐week old recipient mice (WT control and *Gsdmc2–4* ^−/−^ mice) were lethally irradiated by 800 cGy, followed by injection in the tail vein of 5 × 10^6^ bone marrow cells isolated from donor WT mice or *Gsdmc2–4* ^−/−^ mice. Four chimera groups were generated: WT → WT, *Gsdmc2–4* ^−/−^ → WT, WT → *Gsdmc2–4* ^−/−^, and *Gsdmc2–4* ^−/−^ → *Gsdmc2–4* ^−/−^. The transplanted mice were administrated drinking water with 2g L^−1^ neomycin sulfate for 2 weeks. Induction of CRC was conducted 8 weeks after bone marrow reconstitution, as described in the section ‘AOM Plus DSS Induced Colorectal Cancer.’

### Subcutaneous Tumor Cell Graft

Mice at the age of 6–8 weeks were used for subcutaneous tumor cell graft. 2 × 10^5^ MC38 cells or CT26‐luciferase cells stably expressing GSDMC2/3/4, shGSDMC, or control empty vectors were suspended in 100 µL PBS and injected subcutaneously into the right buttock area of C57BL/6 (for MC38 cells), BALB/c (for CT26 cells), or nude mice (for both MC38 and CT26 cells). The growth of CT26‐luciferase cells was monitored twice a week by bioluminescence imaging (IVIS imaging system, PerkinElmer, USA).

### H&E, Immunofluorescence, and Immunohistochemical Staining

Freshly isolated tumor tissues were fixed in 4% paraformaldehyde overnight, washed with PBS once, and then transferred to 70%, 80%, 90%, and straight ethanol consecutively. The samples were embedded in paraffin, sectioned, and stained with hematoxylin‐eosin.

For immunofluorescence assay, the samples were blocked with 3% bovine serum albumin at room temperature for 30 min. The sections were incubated overnight at 4 °C with primary antibodies against E‐cadherin (1:1000 dilution), α‐SMA (1:200 dilution), CXCR2(1:500 dilution), or CD3 (1:1000 dilution). The slides were washed in PBS three times and covered with HRP‐labeled secondary antibody of the corresponding species of the primary antibodies for 1 h at room temperature. After three washes in PBS, the iF488‐Tyramide (1:500) was dropped and incubated at room temperature for 10 min. After incubation, the slides were washed three times in PBS. For CD3 staining, DAPI dye solution was dropped and incubated at room temperature for 10 min away from light, and the slides were sealed with anti‐fluorescence quenching mounting medium. For multicolor immunofluorescence staining, the tissue sections were placed in the citric acid antigen repair buffer, 100 °C for 10 min. The samples were blocked again with 3% bovine serum albumin at room temperature for 30 min. The sections were incubated overnight at 4 °C with the second primary antibodies against CXCL2 (1:200 dilution), CXCL1 (1:200 dilution), GR‐1(1:200 dilution), or GSDMC (1:200 dilution). After washing, the slides were covered with HRP‐labeled corresponding secondary antibody for 1 h at room temperature. After three washes in PBS, the iF555‐Tyramide (1:500) was dropped and incubated at room temperature for 10 min. After washing, the DAPI dye solution was dropped and incubated at room temperature for 10 min away from light. The slides were sealed with anti‐fluorescence quenching mounting medium. Images were acquired by confocal microscope by NIS‐Elements software using the 10×, 20× objective.

For immunohistochemical staining, the samples were blocked with 3% bovine serum albumin at room temperature for 30 min. The sections were incubated overnight at 4 °C with primary antibodies against human GSDMC (1:200 dilution), mouse GSDMC2/3 (1:200 dilution), GR‐1 (1:200 dilution), CD31 (1:200 dilution), and Ki67 (1:200 dilution). The slides were washed in PBS three times and covered with HRP‐labeled secondary antibody of the corresponding species of the primary antibodies for 1 h at room temperature. After three washes, the slides were stained with freshly prepared DAB color developing solution. The slides were washed with distilled water and stained with hematoxylin solution for nuclei. The samples were dehydrated and made transparent by alcohol, n‐butanol, and xylene. The slides were sealed, and the sections were observed under a light microscope.

Staining was quantified using at least five randomly selected 20× fields of view. All staining was quantified using NIH ImageJ analysis software with the same threshold for each set (http://rsb.info.nih.gov/nih‐image/).

### Flow Cytometry

Tumors were dissected and chopped into small pieces and disintegrated with 0.01% w/v Liberase TH and 100 U mL^−1^ DNase I in RPMI 1640 at 37 °C for 30 min. Cells were then filtered through a 40‐µm cell strainer and washed with 5 mL wash buffer (1× PBS with 2 mm EDTA and 0.5% BSA), followed by centrifugation at 200 × *g* for 5 min. Cells were resuspended with 5 mL ACK lysis buffer, holding on ice for 5 min. The cells were washed with 10 mL wash buffer twice. The cells were blocked with CD16/32 antibody for 10 min and stained with fluorescent‐conjugated antibodies: CD45, CD11b, F4/80, GR‐1, and Ly6G for 30 min. The cells were washed, resuspended in PBS containing 1% BSA, and analyzed by BD FACS Aria II flow cytometer (Franklin Lakes, NJ, USA).

### TUNEL Assay

For determination of cell apoptosis, TUNEL assay was performed with the In Situ Cell Death Kit (Roche) according to the manufacturer's instructions. Images were obtained with fluorescence microscope.

### Western Blots Analysis

Tissues or cells were homogenized in RIPA lysis buffer and lyzed on ice for 30 min. Supernatants were collected after centrifugation (12 000 _×_ *g*) at 4 °C for 30 min. Lysates were separated by sodium dodecyl sulfate‐polyacrylamide gel electrophoresis (SDS‐PAGE) and blotted onto a polyvinylidene fluoride membrane. After blocking, the membrane was probed with the indicated primary antibody at 4 °C overnight. The membrane was rinsed and incubated with a horseradish peroxidase conjugated secondary antibody at room temperature for 1 h, followed by visualization using an enhanced chemiluminescence (ECL) detection system. Band intensity was given relative to the corresponding band intensity of GAPDH and was quantified by Image J software.

### qPCR Analysis

Total RNA was reverse transcribed into complementary DNA (cDNA) and analyzed with SYBR Premix Ex Taq (Takara) on 7500 Real‐Time PCR System (Applied Biosystems). Relative mRNA expression level for each gene was assessed by normalization to the expression of the housekeeping gene *Rpl13a*.

Primer list:

mGSDMC1‐F: ATGTCCTACACATTTGACTGGC

mGSDMC1‐R: TTAAACAGGCAGAATTTGGTTGC

mGSDMC2‐F: CTGTGGAATGCTTGTCCGATG

mGSDMC2‐R: CCTCCAGGTCCGTTGATTGG

mGSDMC3‐F: TGCTCAGTGATAGCCAACTCA

mGSDMC3‐R: TGGCTGTAGGATGCTCGTTA

mGSDMC4‐F: TGAGGAGCCTGCCAATCTAAA

mGSDMC4‐R: ATGTGGGGTGCTAGAATCCTT

mCXCL1‐F: ACTGCACCCAAACCGAAGTC

mCXCL1‐R: TGGGGACACCTTTTAGCATCTT

mCXCL2‐F: CCAACCACCAGGCTACAGG

mCXCL2‐R: GCGTCACACTCAAGCTCTG

mCXCL3‐F: ACACCCTACCAAGGGTTGATTTT

mCXCL3‐R: GAGTGGCTATGACTTCTGTCTGG

mCXCL5‐F: GTTCCATCTCGCCATTCATGC

mCXCL5‐R: GCGGCTATGACTGAGGAAGG

mCXCL7‐F: CTCAGACCTACATCGTCCTGC

mCXCL7‐R: GTGGCTATCACTTCCACATCAG

mCXCL15‐F: TCGAGACCATTTACTGCAACAG

mCXCL15‐R: CATTGCCGGTGGAAATTCCTT

mIL‐1β‐F:GAAATGCCACCTTTTGACAGTG

mIL‐1β‐R:TGGATGCTCTCATCAGGACAG

mIL‐6‐F:CTGCAAGAGACTTCCATCCAG

mIL‐6‐R:AGTGGTATAGACAGGTCTGTTGG

mTNFα‐F:CAGGCGGTGCCTATGTCTC

mTNFα‐R:CGATCACCCCGAAGTTCAGTAG

mNOS2‐F:GGAGTGACGGCAAACATGACT

mNOS2‐R:TCGATGCACAACTGGGTGAAC

mRPL13A‐F: GGGCAGGTTCTGGTATTGGAT

mRPL13A‐R: GGCTCGGAAATGGTAGGGG

### RNA Extraction, Library Construction, and Sequencing

Tissues or cells were homogenized and preserved in Trizol reagent, and total RNA was extracted by trichloromethane extraction, isopropanol precipitation, ethanol rinsing, and Rnase‐free water dissolution. The entire mRNAseq library was constructed using the ALFA‐SEQ Directional RNaLib Prep Kit according to the manufacturer's instructions. In brief, mRNA was purified from total RNA using poly‐T oligo‐attached magnetic beads. mRNA fragmentation, cDNA synthesis, blunt ends convertion, ligation of the VAHTS Adapter‐S with a hairpin loop structure, and purification of 150–200 bp fragments were performed according to the manufacturer's instructions. PCR was performed using 2× PfuMax HiFi PCR ProMix (EnzyValley) and primers with index VAHTS Multiplex Oligos Set 4 for Illumina. After purification, the library insert sizes were evaluated on the Qsep400 high‐throughput nucleic acid protein analysis system (Houze Biotechnology Co., Ltd., Hangzhou, China). The clustering of the index‐coded samples was performed on a cBot Cluster Generation System. Then, the library was sequenced on an Illumina Novaseq 6000 platform.

The raw RNA sequencing reads were processed through Fastp (v.0.23.2) for clean reads. The clean reads were mapped to the NCBI Rfam database, and rRNA sequences were removed by Bowtie2 (v2.33). Alignment to the reference genome was performed by Hisat2 (v2.2.1). After that, RSEM (v1.3.3) was applied to obtain the read count for each gene. Differentially expressed genes (FDR ≤ 0.05 and |log2(Fold Change) |≥1) were analyzed using DESeq2 (v1.34.0) among groups. Hierarchical clustering was performed using DEGs with the highest variability across the samples.

### Enzyme‐Linked Immunosorbent Assay

For quantification of HMGB1 release in CRC interstitial fluid, 100 mg of freshly isolated tumors or colon tissues was homogenized mechanically in PBS containing 1% NP‐40 and a complete protease inhibitor cocktail. For quantification of HMGB1 release in culture supernatants, after indicated treatments without FBS, supernatants were harvested by centrifugation at 4 °C. The protein level of HMGB1 was measured with HMGB1 ELISA Kits according to the manufacturer's instructions.

For quantification of CXCL1 and CXCL2, tumors were freshly collected and subjected to RIPA lysis with a complete protease inhibitor cocktail. The protein levels of CXCL1 and CXCL2 were measured with corresponding ELISA kits according to the manufacturer's instructions.

### MDSCs Isolation and In Vitro Migration Assay

MDSCs were isolated from the spleens of AOM‐DSS induced CRC mice using a Mouse Myeloid‐Derived Suppressor Cell Isolation Kit. MDSCs (1 × 10^5^ cells per well) were seeded in the top chamber of a 24‐well transwell. To obtain conditioned media (CM) from tumor tissue, 100 mg freshly isolated tumors was cut into ≈1 mm^3^ pieces; the tumor slices were cultured in hypoxia chamber with DMEM (low glucose) supplemented with 10% FBS, 1× Insulin‐Transferrin‐Selenium, 1× GlutaMAX, and penicillin (100 U mL^−1^) and streptomycin (100 µg mL^−1^) for 48 h. After culturing, the supernatants were collected. CM was diluted with complete medium and added to the bottom layer of the transwell for MDSC migration assay. After 4 h incubation, cells that had completely migrated to the bottom chamber were counted. To validate in vitro, the CXCL2‐mediated migration of MDSCs, 2 µg mL^−1^ mouse CXCL2 neutralizing Antibody (R&D, MAB452) was added.

### Statistics

GraphPad Software was used to perform statistical analysis and graph development. Data were presented as the mean ± SEM. A two‐tailed Student's *t‐*test was used. Survival curves were presented using the Kaplan–Meier method, and significance was calculated by log‐rank (Mantel‐Cox) test. *p* values < 0.05 were considered statistically significant. *p < 0.05, **p < 0.01, and ***p < 0.001.

### Ethics Approval and Consent to Participate

All animal experiments were approved by the Animal Care and Use Committee of Guangdong Medical University (approval ID: GDY2302376). The use of human specimens in this study was approved by the Institutional Research Ethics Committee of Shenzhen Longhua District Central Hospital (approval ID: 2024‐035‐01).

## Conflict of Interest

The authors declare no conflict of interest.

## Author Contributions

H.G. and Peng. C. designed and performed experiments, analyzed and interpreted data, and wrote the manuscript. Y.Y. and W.S. edited the manuscript. G.S. interpreted data and revised the manuscript. W.L., Z.X., W.H., K.L., Pei. C., and S.L. provided technical support. M.C. and Peng C. supervised experiments.

## Supporting information



Supporting Information

## Data Availability

Sequencing data have been deposited in the National Center for Biotechnology Information repository under accession code PRJNA1128794. Data, analytical methods, and research materials in this article will be available to other researchers from the corresponding authors upon reasonable request.

## References

[1] M. Schmitt , F. R. Greten , Nat. Rev. Immunol. 2021, 21, 653. 33911231 10.1038/s41577-021-00534-x

[2] X. Li , Y. Ma , J. Wu , M. Ni , A. Chen , Y. Zhou , W. Dai , Z. Chen , R. Jiang , Y. Ling , Q. Yao , W. Chen , Drug Resistance Updates 2023, 67, 100930. 36736043 10.1016/j.drup.2023.100930

[3] E. R. Fearon , Annu. Rev. Pathol.:Mech. Dis. 2011, 6, 479. 10.1146/annurev-pathol-011110-130235 21090969

[4] Y. Di , X. Jing , K. Hu , X. Wen , L. Ye , X. Zhang , J. Qin , J. Ye , R. Lin , Z. Wang , W. He , Drug Resistance Updates 2023, 66, 100909. 36525936 10.1016/j.drup.2022.100909

[5] V. Kumar , S. Patel , E. Tcyganov , D. I. Gabrilovich , Trends Immunol. 2016, 37, 208. 26858199 10.1016/j.it.2016.01.004 PMC4775398

[6] X. Jing , F. Yang , C. Shao , K. Wei , M. Xie , H. Shen , Y. Shu , Mol. Cancer 2019, 18, 157. 31711497 10.1186/s12943-019-1089-9 PMC6844052

[7] W. H. Chang , A. G. Lai , Cancer Lett. 2020, 487, 34. 32470490 10.1016/j.canlet.2020.05.011

[8] B. Wang , Q. Zhao , Y. Zhang , Z. Liu , Z. Zheng , S. Liu , L. Meng , Y. Xin , X. Jiang , J. Exp. Clin. Cancer Res. 2021, 40, 24. 33422072 10.1186/s13046-020-01820-7 PMC7796640

[9] C. A. Corzo , T. Condamine , L. Lu , M. J. Cotter , J.‐I. Youn , P. Cheng , H.‐I. Cho , E. Celis , D. G. Quiceno , T. Padhya , T. V. McCaffrey , J. C. McCaffrey , D. I. Gabrilovich , J. Exp. Med. 2010, 207, 2439. 20876310 10.1084/jem.20100587 PMC2964584

[10] V. Kumar , P. Cheng , T. Condamine , S. Mony , L. R. Languino , J. C. McCaffrey , N. Hockstein , M. Guarino , G. Masters , E. Penman , F. Denstman , X. Xu , D. C. Altieri , H. Du , C. Yan , D. I. Gabrilovich , Immunity 2016, 44, 303. 26885857 10.1016/j.immuni.2016.01.014 PMC4759655

[11] D. K.‐C. Chiu , A. P.‐W. Tse , I. M.‐J. Xu , J. Di Cui , R. K.‐Ho Lai , L. L. Li , H.‐Y Koh , F. H.‐C. Tsang , L. L. Wei , C.‐M. Wong , I. O.‐L. Ng , C. C.‐L. Wong , Nat. Commun. 2017, 8, 517. 28894087 10.1038/s41467-017-00530-7 PMC5593860

[12] J. Shi , Y. Zhao , K. Wang , X. Shi , Y. Wang , H. Huang , Y. Zhuang , T. Cai , F. Wang , F. Shao , Nature 2015, 526, 660. 26375003 10.1038/nature15514

[13] C. B. Ryder , H. C. Kondolf , M. E. O'Keefe , B. Zhou , D. W. Abbott , J. Mol. Biol. 2022, 434, 167183. 34358546 10.1016/j.jmb.2021.167183 PMC8810912

[14] E. E. Elias , B. Lyons , D. A. Muruve , Nat. Rev. Nephrol. 2023, 19, 337. 36596918 10.1038/s41581-022-00662-0

[15] J. Wei , Z. Xu , X. Chen , X. Wang , S. Zeng , L. Qian , X. Yang , C. Ou , W. Lin , Z. Gong , Y. Yan , Mol. Med. Rep. 2020, 21, 360. 31939622 10.3892/mmr.2019.10837 PMC6896373

[16] J. Hou , R. Zhao , W. Xia , C.‐W. Chang , Y. You , J.‐M. Hsu , L. Nie , Y. Chen , Y.‐C. Wang , C. Liu , W.‐J. Wang , Y. Wu , B. Ke , J. L. Hsu , K. Huang , Z. Ye , Y. Yang , X. Xia , Y. Li , C.‐W. Li , B. Shao , J. A. Tainer , M.‐C. Hung , Nat. Cell Biol. 2020, 22, 1264. 32929201 10.1038/s41556-020-0575-z PMC7653546

[17] G. Privitera , N. Rana , A. Armuzzi , T. T. Pizarro , Nat. Rev. Gastroenterol. Hepatol. 2023, 20, 366. 36781958 10.1038/s41575-023-00743-w PMC10238632

[18] J.‐Y. Zhang , B. Zhou , R.‐Y. Sun , Y.‐L. Ai , K. Cheng , F.‐N. Li , B.‐R. Wang , F.‐J. Liu , Z.‐H. Jiang , W.‐J. Wang , D. Zhou , H.‐Z. Chen , Q. Wu , Cell Res. 2021, 31, 980. 34012073 10.1038/s41422-021-00506-9 PMC8410789

[19] S. Wang , C.‐W. Chang , J. Huang , S. Zeng , X. Zhang , M.‐C. Hung , J. Hou , J. Clin. Invest. 2024, 134, 166841. 10.1172/JCI166841 PMC10760963 37883181

[20] R. Xi , J. Montague , X. Lin , C. Lu , W. Lei , K. Tanaka , Y. V. Zhang , X. Xu , X. Zheng , X. Zhou , J. F. Urban, Jr. , K. Iwatsuki , R. F. Margolskee , I. Matsumoto , M. Tizzano , J. Li , P. Jiang , Proc. Natl. Acad. Sci. U. S. A. 2021, 118. 10.1073/pnas.2026307118 PMC8325246 34290141

[21] M. Zhao , K. Ren , X. Xiong , Y. Xin , Y. Zou , J. C. Maynard , A. Kim , A. P. Battist , N. Koneripalli , Y. Wang , Q. Chen , R. Xin , C. Yang , R. Huang , J. Yu , Z. Huang , Z. Zhang , H. Wang , D. Wang , Y. Xiao , O. C. Salgado , N. N. Jarjour , K. A. Hogquist , X. S. Revelo , A. L. Burlingame , X. Gao , J. von Moltke , Z. Lin , H.‐B. Ruan , Immunity 2022, 55, 623. 35385697 10.1016/j.immuni.2022.03.009 PMC9109499

[22] M. Miguchi , T. Hinoi , M. Shimomura , T. Adachi , Y. Saito , H. Niitsu , M. Kochi , H. Sada , Y. Sotomaru , T. Ikenoue , K. Shigeyasu , K. Tanakaya , Y. Kitadai , K. Sentani , N. Oue , W. Yasui , H. Ohdan , PLoS One 2016, 11, 0166422. 10.1371/journal.pone.0166422 PMC5105946 27835699

[23] E. Zapletal , T. Vasiljevic , P. Busson , T. M. Glavan , Int. J. Mol. Sci. 2023, 24, 5278. 36982351 10.3390/ijms24065278 PMC10049335

[24] L. Zhu , X. Zhu , Y. Wu , Biomolecules 2022, 12, 580. 35454171 10.3390/biom12040580 PMC9028125

[25] J. E. Bader , K. Voss , J. C. Rathmell , Mol. Cell 2020, 78, 1019. 32559423 10.1016/j.molcel.2020.05.034 PMC7339967

[26] H. Guo , D. Pétrin , Y. Zhang , C. Bergeron , C. G. Goodyer , A. C. LeBlanc , Cell Death Differ. 2006, 13, 285. 16123779 10.1038/sj.cdd.4401753

[27] V. Kaushal , R. Dye , P. Pakavathkumar , B. Foveau , J. Flores , B. Hyman , B. Ghetti , B. H. Koller , A. C. LeBlanc , Cell Death Differ. 2015, 22, 1676. 25744023 10.1038/cdd.2015.16 PMC4563782

[28] Z. Ma , K. Li , P. Chen , J. Pan , X. Li , G. Zhao , Biol. Pharm. Bull. 2020, 43, 1481. 32999158 10.1248/bpb.b20-00050

[29] O. Zavidij , N. J. Haradhvala , T. H. Mouhieddine , R. Sklavenitis‐Pistofidis , S. Cai , M. Reidy , M. Rahmat , A. Flaifel , B. Ferland , N. K. Su , M. P. Agius , J. Park , S. Manier , M. Bustoros , D. Huynh , M. Capelletti , B. Berrios , C.‐J. Liu , M. X. He , E. Braggio , R. Fonseca , Y. E. Maruvka , J. L. Guerriero , M. Goldman , E. M. Van Allen , S. A. McCarroll , J. Azzi , G. Getz , I. M. Ghobrial , Nat. Cancer 2020, 1, 493. 33409501 10.1038/s43018-020-0053-3 PMC7785110

[30] J. Peng , B.‐F. Sun , C.‐Y. Chen , J.‐Y. Zhou , Y.‐S. Chen , H. Chen , L. Liu , D. Huang , J. Jiang , G.‐S. Cui , Y. Yang , W. Wang , D. Guo , M. Dai , J. Guo , T. Zhang , Q. Liao , Y. Liu , Y.‐L. Zhao , D.‐L. Han , Y. Zhao , Y.‐G. Yang , W. Wu , Cell Res. 2019, 29, 725. 31273297 10.1038/s41422-019-0195-y PMC6796938

[31] Q. Song , G. A. Hawkins , L. Wudel , P.‐C. Chou , E. Forbes , A. K. Pullikuth , L. Liu , G. Jin , L. Craddock , U. Topaloglu , G. Kucera , S. O'Neill , E. A. Levine , P. Sun , K. Watabe , Y. Lu , M. A. Alexander‐Miller , B. Pasche , L. D. Miller , W. Zhang , Cancer Med. 2019, 8, 3072. 31033233 10.1002/cam4.2113 PMC6558497

[32] A. T. Yeo , S. Rawal , B. Delcuze , A. Christofides , A. Atayde , L. Strauss , L. Balaj , V. A. Rogers , E. J. Uhlmann , H. Varma , B. S. Carter , V. A. Boussiotis , A. Charest , Nat. Immunol. 2022, 23, 971. 35624211 10.1038/s41590-022-01215-0 PMC9174057

[33] W. Liao , M. J. Overman , A. T. Boutin , X. Shang , D. Zhao , P. Dey , J. Li , G. Wang , Z. Lan , J. Li , M. Tang , S. Jiang , X. Ma , P. Chen , R. Katkhuda , K. Korphaisarn , D. Chakravarti , A. Chang , D. J. Spring , Q. Chang , J. Zhang , D. M. Maru , D. Y. Maeda , J. A. Zebala , S. Kopetz , Y. A. Wang , R. A. DePinho , Cancer Cell 2019, 35, 559. 30905761 10.1016/j.ccell.2019.02.008 PMC6467776

[34] L. Seifert , G. Werba , S. Tiwari , N. N. Giao Ly , S. Alothman , D. Alqunaibit , A. Avanzi , R. Barilla , D. Daley , S. H. Greco , A. Torres‐Hernandez , M. Pergamo , A. Ochi , C. P. Zambirinis , M. Pansari , M. Rendon , D. Tippens , M. Hundeyin , V. R. Mani , C. Hajdu , D. Engle , G. Miller , Nature 2016, 532, 245. 27049944 10.1038/nature17403 PMC4833566

[35] S. A. Lasser , F. G. Ozbay Kurt , I. Arkhypov , J. Utikal , V. Umansky , Nat. Rev. Clin. Oncol. 2024, 21, 147. 38191922 10.1038/s41571-023-00846-y

[36] K. Li , H. Shi , B. Zhang , X. Ou , Q. Ma , Y. Chen , P. Shu , D. Li , Y. Wang , Signal Transduction Targeted Ther. 2021, 6, 362. 10.1038/s41392-021-00670-9 PMC8497485 34620838

[37] F. Veglia , E. Sanseviero , D. I. Gabrilovich , Nat. Rev. Immunol. 2021, 21, 485. 33526920 10.1038/s41577-020-00490-y PMC7849958

[38] H. Katoh , D. Wang , T. Daikoku , H. Sun , S. K. Dey , R. N. DuBois , Cancer Cell 2013, 24, 631. 24229710 10.1016/j.ccr.2013.10.009 PMC3928012

[39] R. K. Boyapati , A. G. Rossi , J. Satsangi , G.‐T. Ho , Mucosal Immunol. 2016, 9, 567. 26931062 10.1038/mi.2016.14

[40] F. Castro , A. P. Cardoso , R. M. Gonçalves , K. Serre , M. J. Oliveira , Front. Immunol. 2018, 9, 847. 29780381 10.3389/fimmu.2018.00847 PMC5945880

[41] A. Montfort , C. Colacios , T. Levade , N. Andrieu‐Abadie , N. Meyer , B. Ségui , Front. Immunol. 2019, 10, 1818. 31417576 10.3389/fimmu.2019.01818 PMC6685295

[42] J. Du , R. Sarkar , Y. Li , L. He , W. Kang , W. Liao , W. Liu , T. Nguyen , L. Zhang , Z. Deng , U. Dougherty , S. S. Kupfer , M. Chen , J. Pekow , M. Bissonnette , C. He , Y. C. Li , Dev. Cell 2022, 57, 1976. 35917813 10.1016/j.devcel.2022.07.006 PMC9398964

[43] C. Groth , L. Arpinati , M. E. Shaul , N. Winkler , K. Diester , N. Gengenbacher , R. Weber , I. Arkhypov , S. Lasser , V. Petrova , H. G. Augustin , P. Altevogt , J. Utikal , Z. G. Fridlender , V. Umansky , Cancers 2021, 13, 726. 33578808 10.3390/cancers13040726 PMC7916588

[44] X. Song , H. Gao , Y. Lin , Y. Yao , S. Zhu , J. Wang , Y. Liu , X. Yao , G. Meng , N. Shen , Y. Shi , Y. Iwakura , Y. Qian , Immunity 2014, 40, 140. 24412611 10.1016/j.immuni.2013.11.018

