# Supplementary material for: Caspase‐6/Gasdermin C‐Mediated Tumor Cell Pyroptosis Promotes Colorectal Cancer Progression Through CXCL2‐Dependent Recruitment of Myeloid‐Derived Suppressor Cells
Source: Adv Sci (Weinh). 2025 Apr 11;12(20):2411375. doi: 10.1002/advs.202411375 (PMC12120772; doi:10.1002/advs.202411375)
Supplement: Supplementary file 1 — Supporting Information [file ADVS-12-2411375-s001.docx]

**Supplementary Figures**

**Figure S1**

**
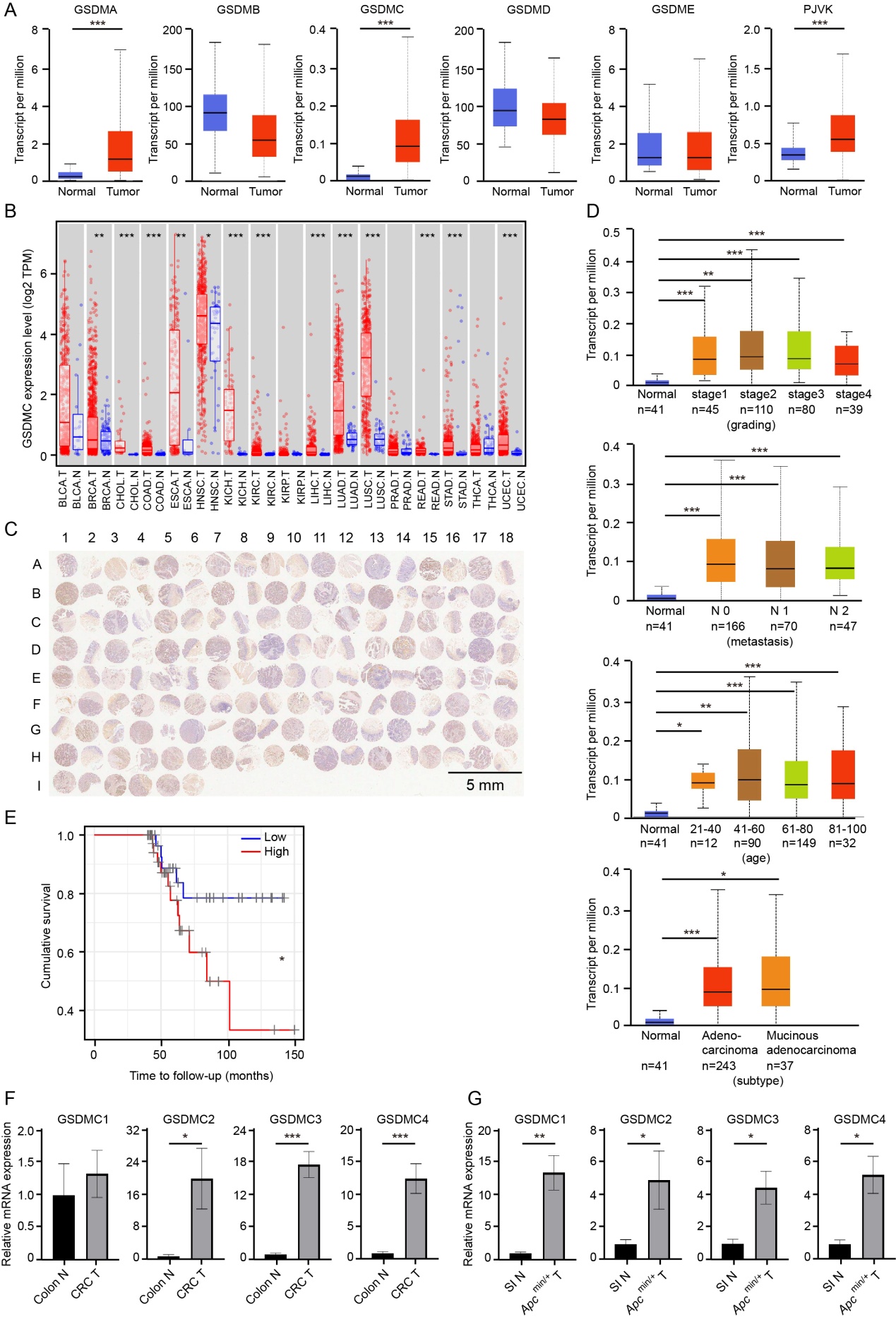
**

**Figure S1. GSDMC is upregulated in human colorectal cancer.**

(A) Transcriptional expression of GSDM family members in colorectal adenocarcinoma from The Cancer Genome Atlas (TCGA) dataset.

(B) Relative GSDMC expression in tumor tissues compared with adjacent normal tissues including BLCA, BRCA, CHOL, COAD, ESCA, HNSC, KICH, KIRP, KIRC, LIHC, LUAD, LUSC, PRAD, READ, STAD, THCA, UCEC from TCGA dataset. BLCA: Bladder Urothelial Carcinoma, BRCA: Breast invasive carcinoma, CHOL: Cholangiocarcinoma, COAD: Colon adenocarcinoma, ESCA: Esophageal carcinoma, HNSC: Head and Neck squamous cell carcinoma, KICH: Head and Neck squamous cell carcinoma, KIRC: Kidney renal clear cell carcinoma, KIRP: Kidney renal papillary cell carcinoma, LIHC: Liver hepatocellular carcinoma, LUAD: Lung adenocarcinoma, LUSC: Lung squamous cell carcinoma, PRAD: Prostate adenocarcinoma, READ: Rectum adenocarcinoma, STAD: Stomach adenocarcinoma, THCA: Stomach adenocarcinoma, UCEC: Uterine Corpus Endometrial Carcinoma.

(C) Immunohistochemical staining of GSDMC in human CRC microarray (75 paired human CRC and adjacent normal tissues ).

(D) Transcriptional GSDMC expression in human CRC subgroups by fractional analysis according to tumor stage, metastasis, subtype, and patients’ age from TCGA CRC dataset.

(E) High GSDMC expression is associated with overall survival by analyzing TCGA CRC dataset. (likelihood ratio test, p < 0.05. Split percentage of patients: 45%. Survival time: >52 months)

(F-G) Quantitative mRNA expression of GSDMC1-4 in AOM-DSS induced mouse CRC tissues (E), and intestinal tumors from *Apc*^min/+^ mice (F).

Data are representative the mean ± SEM in A, B, D, F, and G. *p < 0.05, **p < 0.01, ***p < 0.001 by Student’s t test. SI: small intestine, N: normal, T: tumor.

**Figure S2**

**
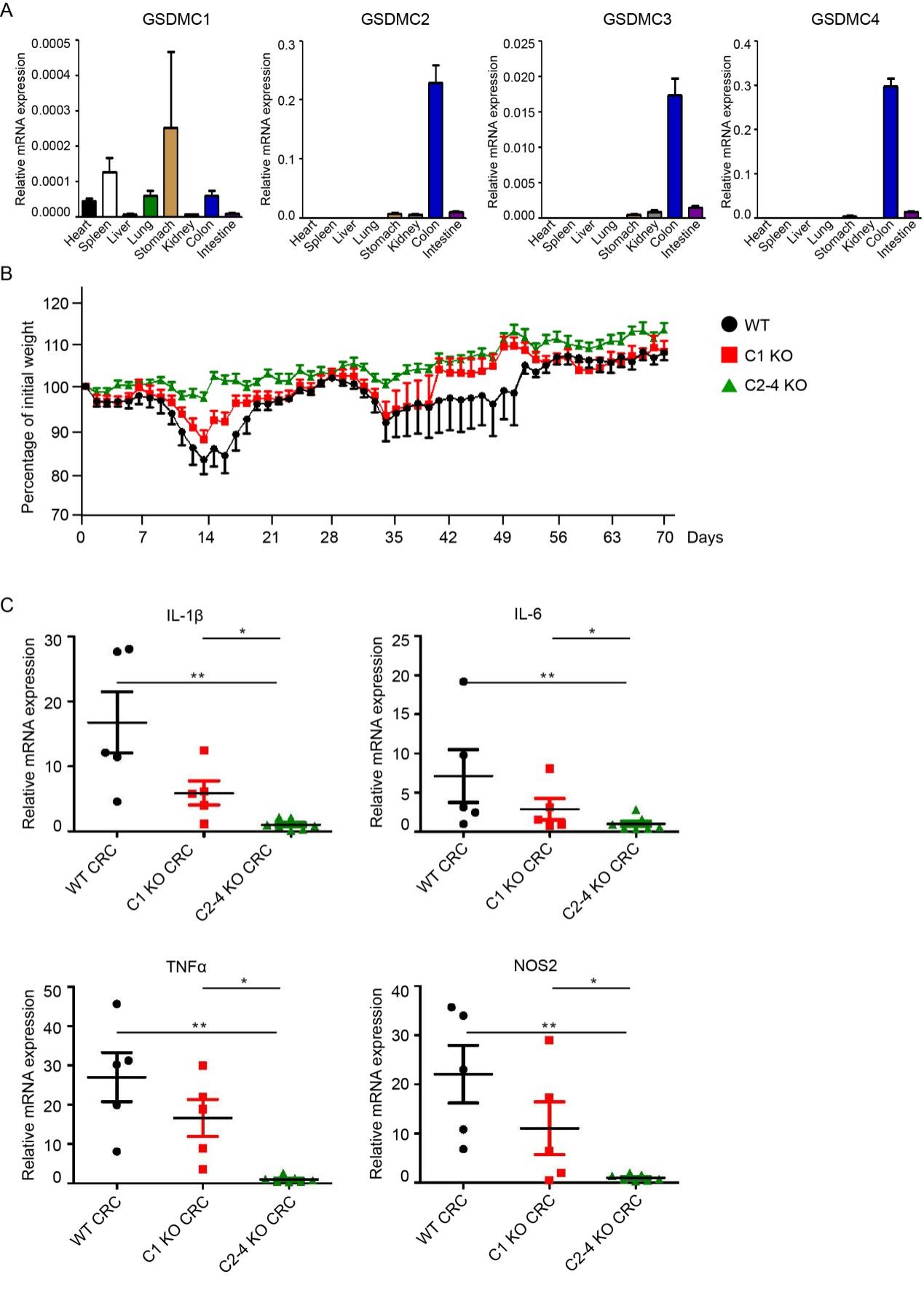
**

**Figure S2. GSDMC2-4 are expressed in mouse colon and promote AOM-DSS induced CRC.**

(A) Quantitative mRNA expression of mouse GSDMC1-4 in heart, spleen, liver, lung, stomach, colon, intestine tissues.

(B) Record of body weight in the course of AOM-DSS induced CRC model.

(C) Quantitative mRNA expression of IL-1β, IL-6, TNFα, and NOS2 in CRC tissues from WT (n=5), *C1* KO (n=5), and *C2-4* KO (n=7) mice.

Data are representative of at least three independent experiments (mean ± SEM in A -C). *p < 0.05, **p < 0.01 by Student’s t test.

**Figure S3**

**
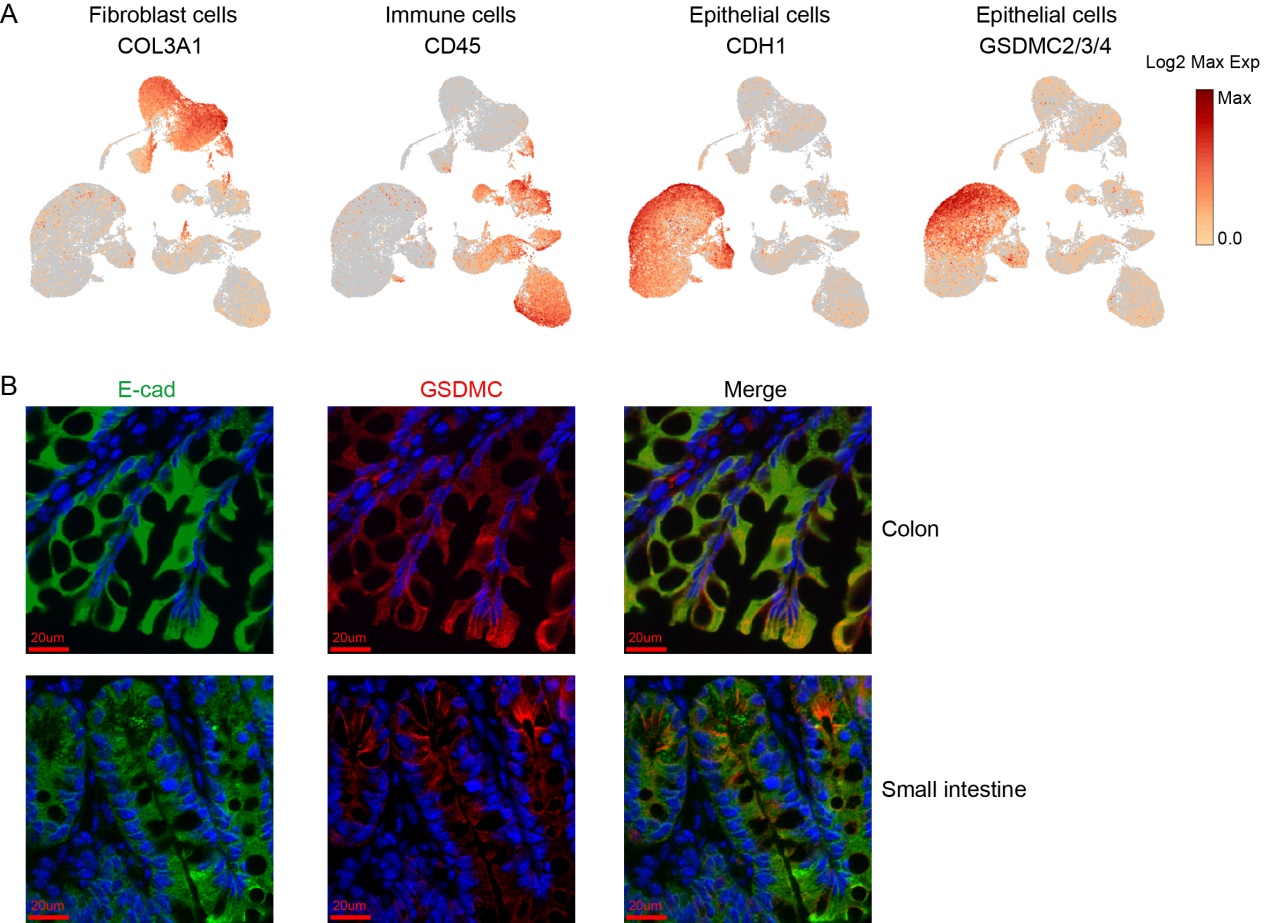
**

**Figure S3. GSDMC is expressed in intestinal epithelial cells.**

(A) UMAP visualization shows the expression of marker genes (*Col3a1*, *Cd45*, *Cdh1*) and *Gsdmc2-4* for the cell types as indicated.

(B) Immunofluorescence staining of E-cad and GSDMC in mouse colon and small intestine. Green for positive E-cad staining, red for positive GSDMC staining and Blue for nucleus.

**Figure S4**

**
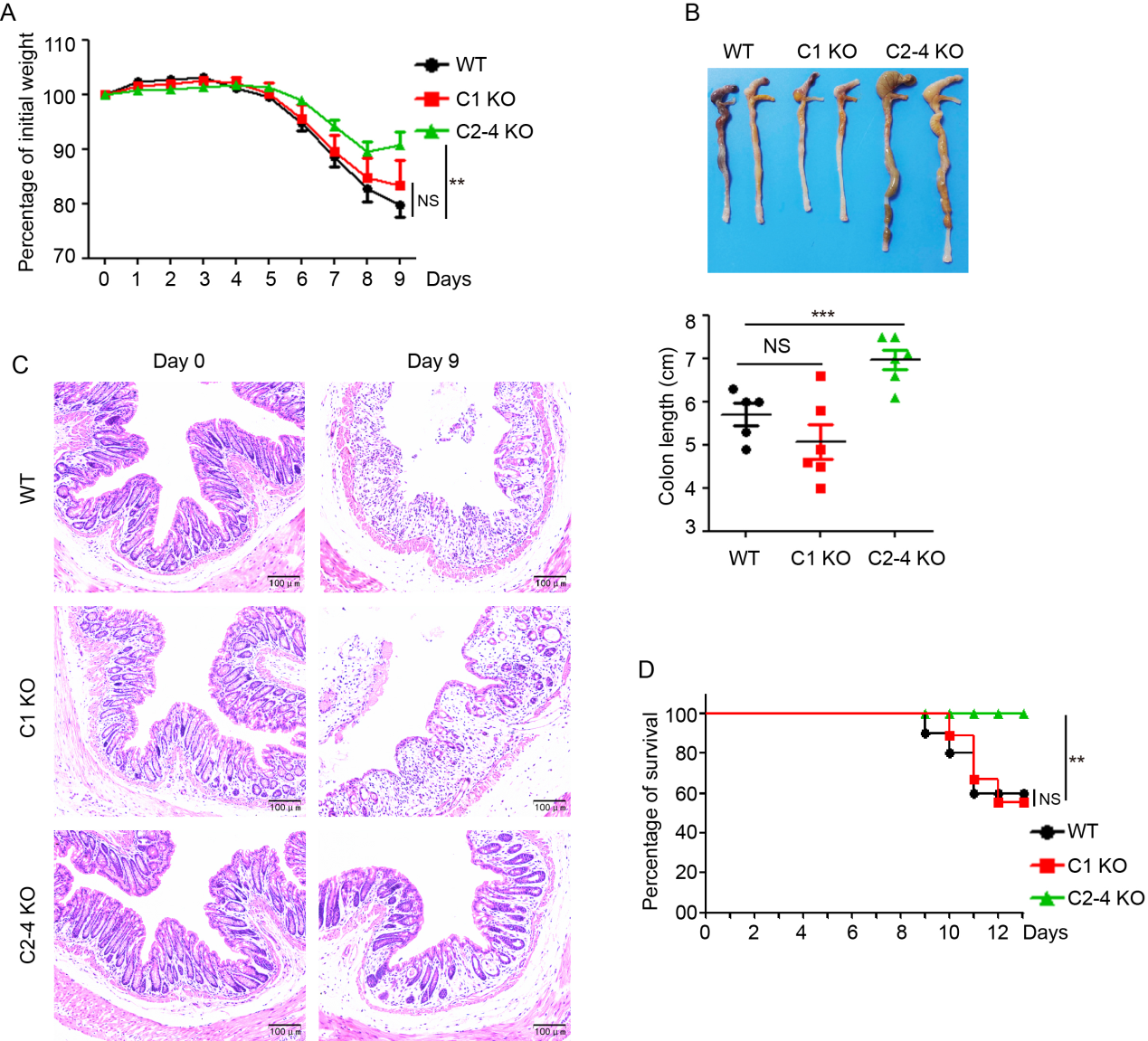
**

**Figure S4. GSDMC2-4 promote DSS induced mouse colitis.**

(A) Record of body weight in the course of DSS induced colitis. (n=5-6 for each group).

(B) Macroscopic view of the representative colons from WT, *C1* KO, and *C2-4* KO mice in DSS induced colitis model. Scatter plots represent the length of colons. (n=5-6 for each group)

(C) H&E histology showing representative colons from WT, *C1* KO, and *C2-4* KO mice in DSS induced colitis model at day 0 and day 9 after DSS administration.

(D) Survival of WT (n=10), *C1* KO (n=9), and *C2-4* KO (n=10) mice in DSS induced colitis model.

Data are representative of at least three independent experiments (mean ± SEM in A and B). **p < 0.01, ***p < 0.001 by Student’s t test. NS: No significance.

**Figure S5**


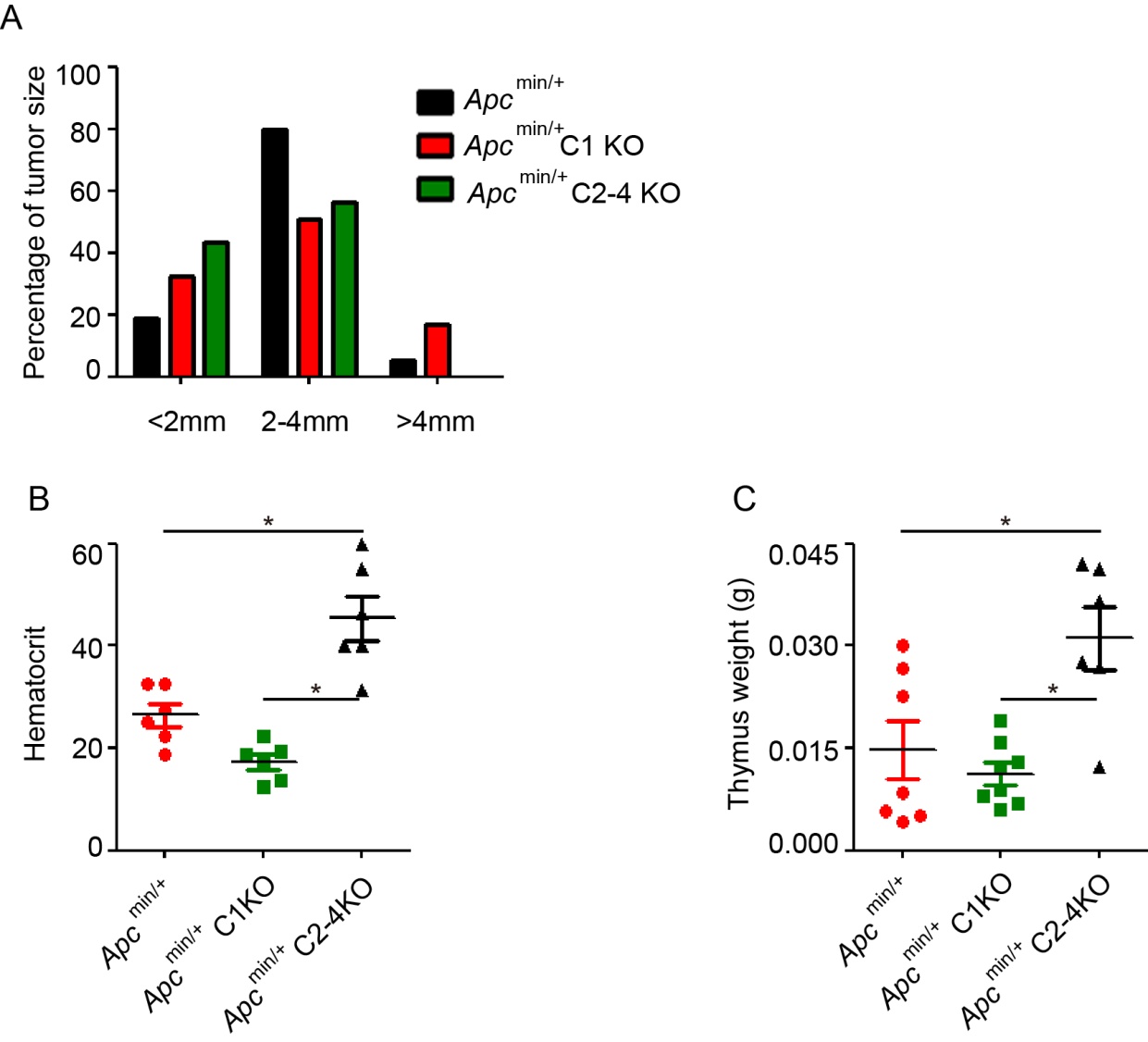


**Figure S5. GSDMC2-4 promote spontaneous intestinal cancer development in *Apc^min/+^* mice.**

(A) Histogram showing the size distribution of small intestines tumors from 20-week old *Apc^min/+^* (n=8), *Apc^min/+^ C1* KO (n=8) and *Apc^min/+^ C2-4* KO (n=6) mice.

(B and C) Hematocrit (B) and thymus weight (C) of mice as indicated in (A) (n=6-7 for each group).

Data represent the mean ± SEM in (B)-(C). *p < 0.05 by Student’s t test.

**Figure S6**


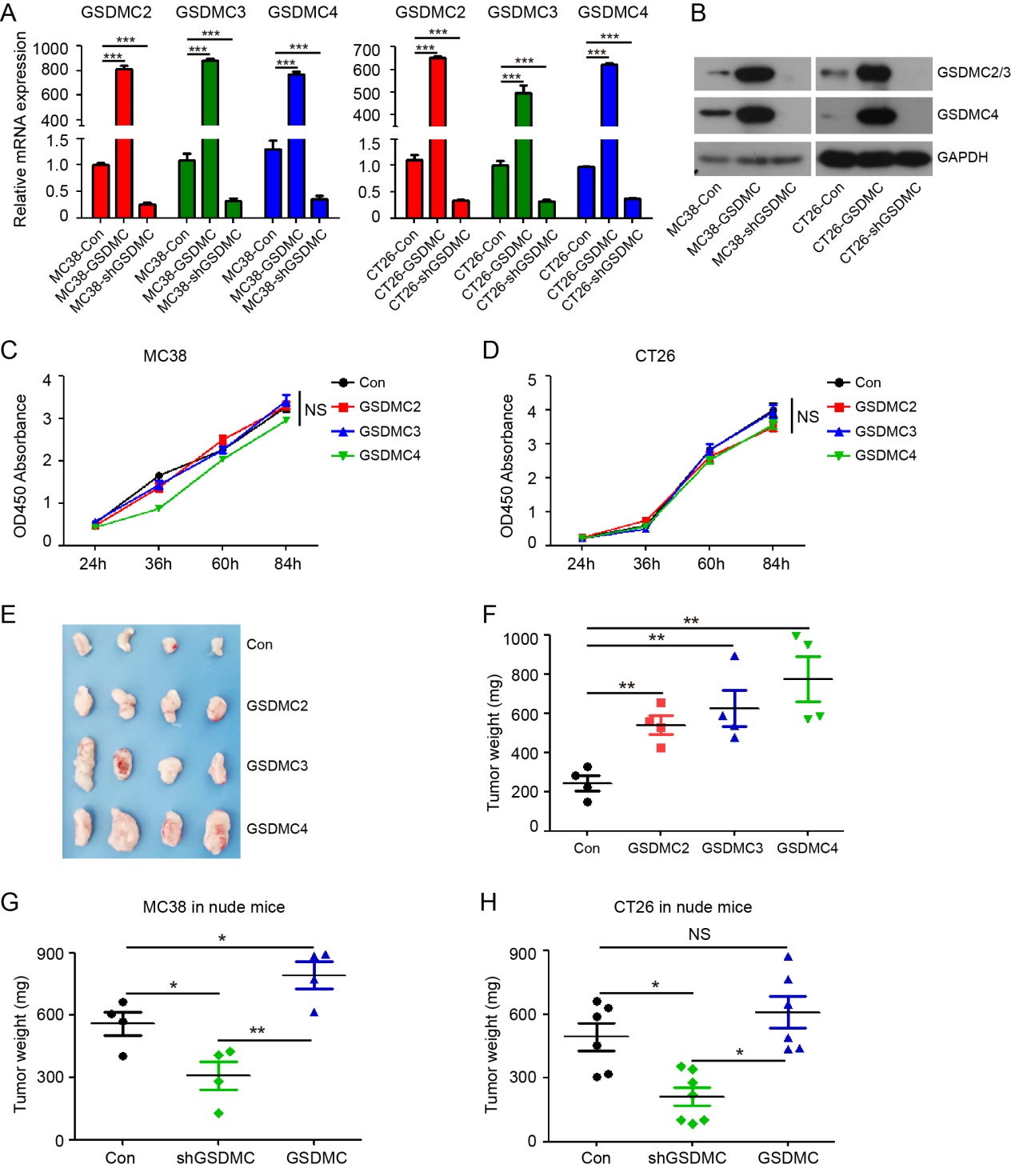


**Figure S6. GSDMC2-4 promote colorectal cancer cell proliferation *in vivo***

(A and B) Quantitative mRNA (A) and protein (B) expression of GSDMC2-4 in MC38 or CT26 cells stably expressing GSDMC2-4, GSDMC shRNA, or control empty vectors.

(C and D) *In vitro* cell proliferation was assessed using CCK-8 method.

(E and F) Macroscopic view of the representative tumors (E) and tumor weight (F) of MC38 cells stably expressing GSDMC2, GSDMC3, GSDMC4, or control empty vectors in the subcutaneous graft model.

(G and H) The tumor weight of MC38 or CT26 cells and their derivates subcutaneously grafted in nude mice.

Data are representative of at least three independent experiments (mean ± SEM in A, C, D, and F-H). *p < 0.05, **p < 0.01, ***p < 0.001 by Student’s t test. NS: No significance.

**Figure S7**

**
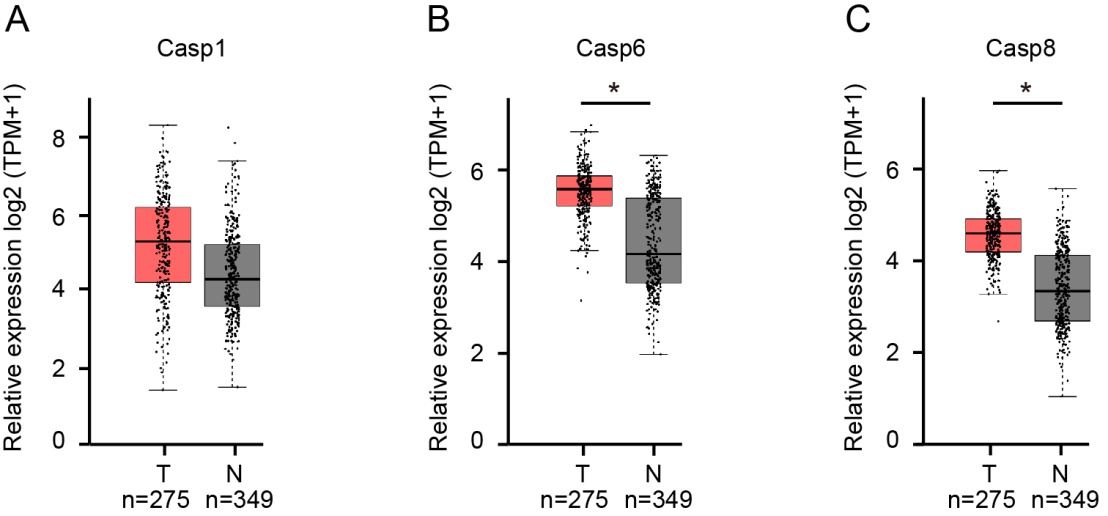
**

**Figure S7. Caspase-1, -6, and -8 expression in human CRC.**

(A-C) Transcriptional expression of Caspase-1 (A), Caspase-6 (B), and Caspase-8 (C) in human CRC from TCGA CRC dataset. Data represent the mean ± SEM in (A)-(C). *p < 0.05 by Student’s t test.

**Figure S8**

**
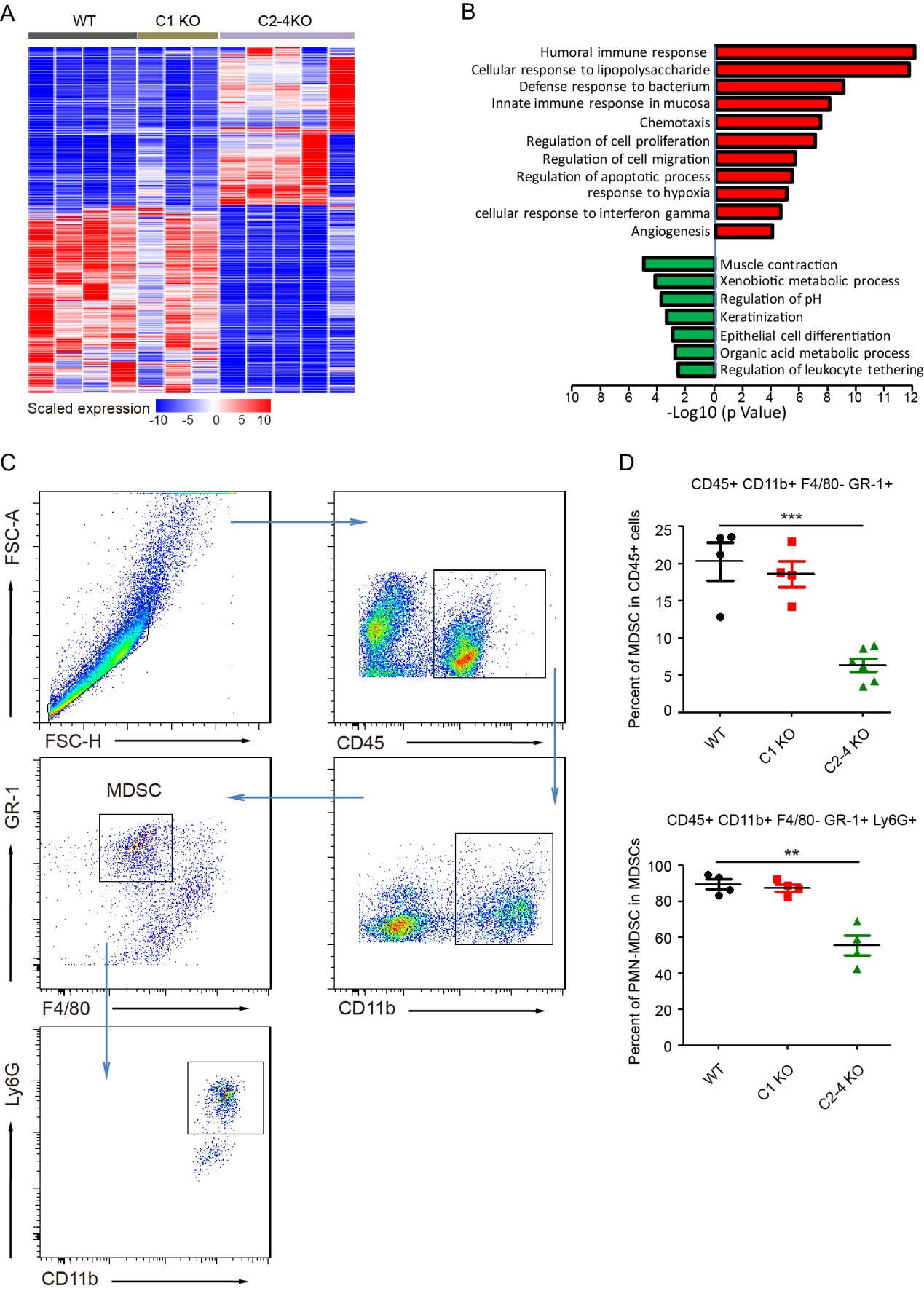
**

**Figure S8. WT and *Gsdmc2-4* deficient CRC tissues showed different gene expression patterns.**

(A) Heatmap dipicting the total differentially expressed genes between WT, *C1* KO, *C2-4* KO CRC tissues (FDR≤0.05 and |log2(Fold Change) |≥1).

(B) KEGG analysis of DEGs upregulated in WT CRC tissues reveals top enriched gene signatrue pathways.

(C-D) Flow cytometry analysis of MDSCs (CD45^+^ CD11b^+^ F4/80^-^ GR-1^+^) and PMN-MDSCs (CD45^+^ CD11b^+^ F4/80^-^ GR-1^+^ Ly6G^+^) in WT, *C1* KO, *C2-4* KO CRC tissues. Data represent the mean ± SEM in D. **p < 0.01, ***p < 0.001 by Student’s t test.

**Figure S9**

**
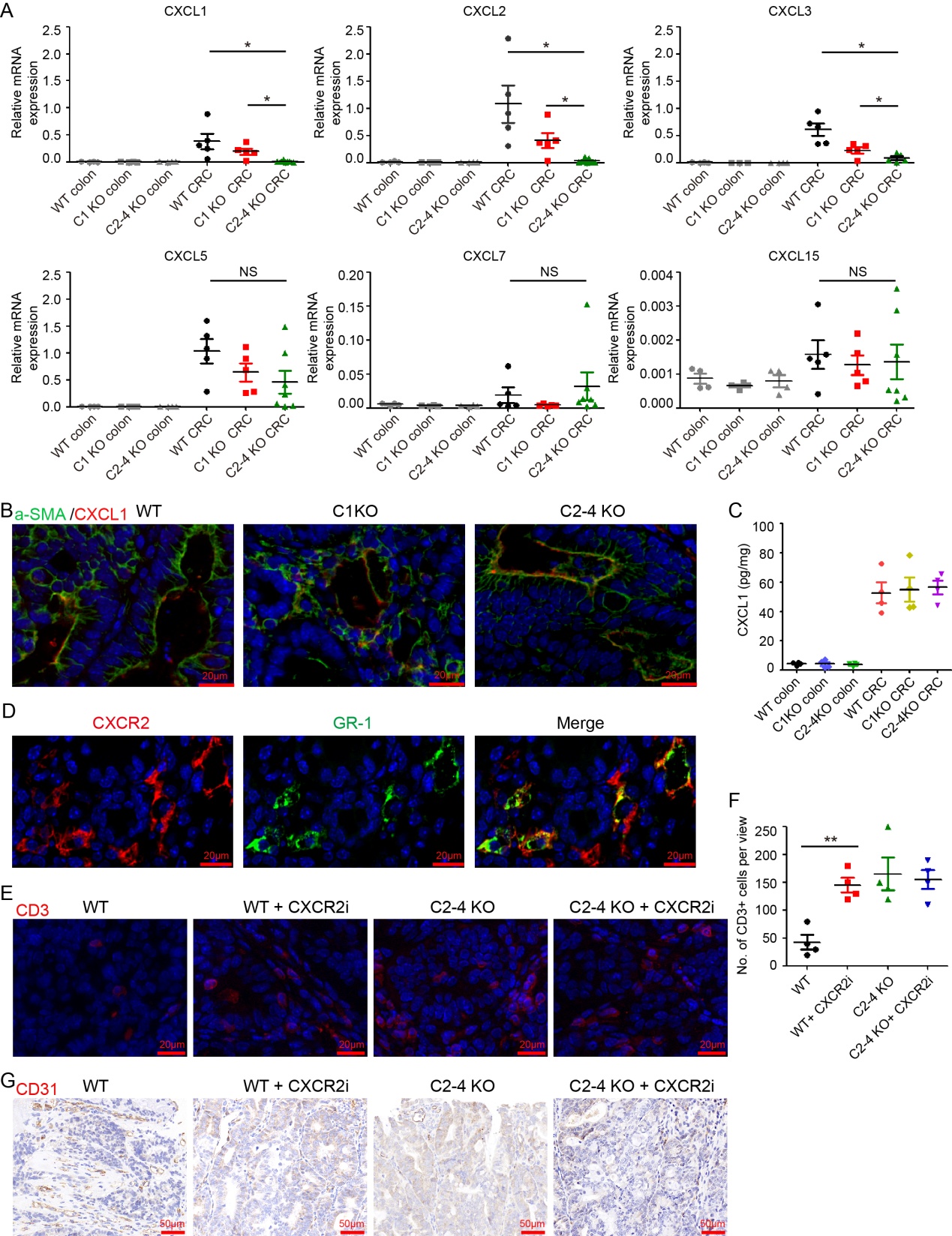
**

**Figure S9. CXCR2 inhibitor increases intratumoral T cell infiltration in AOM-DSS induced CRC.**

(A) Quantitative mRNA expression of *CXCL1*, *CXCL2*, *CXCL3*, *CXCL5*, *CXCL7*, and *CXCL15* in colon and CRC tissues from WT, *C1* KO and *C2-4* KO mice.

(B) Immunofluorescence staining of α-SMA and CXCL1 in colorectal tumors from WT, *C1* KO and *C2-4* KO mice of AOM-DSS induced CRC model.

(C) Colorectal tumors from WT, *C1* KO and *C2-4* KO mice of AOM-DSS induced CRC model were weighted and subjected to RIPA lysis with a complete protease inhibitor cocktail. The protein level of CXCL1 was measured with corresponding ELISA kits. (n=4).

(D) Immunofluorescence staining of CXCR2, and GR-1 in colorectal tumors.

(E) Immunofluorescence staining of CD3 in AOM-DSS induced CRC tissues from WT and *C2-4* KO mice administrated with or without CXCR2 inhibitors.

(F) Number of CD3^+^ cells were counted in immunofluorescence staining slides in 20x magnification fields. n=4 for each group.

(G) Immunohistochemical staining of CD31 in AOM-DSS induced CRC tissues from WT and *C2-4* KO mice administrated with or without CXCR2 inhibitors.

Data are representative of at least two independent experiments (mean ± SEM in A, C and F). *p < 0.05, **p < 0.01 by Student’s t test. NS: No significance.

**Figure S10**

**
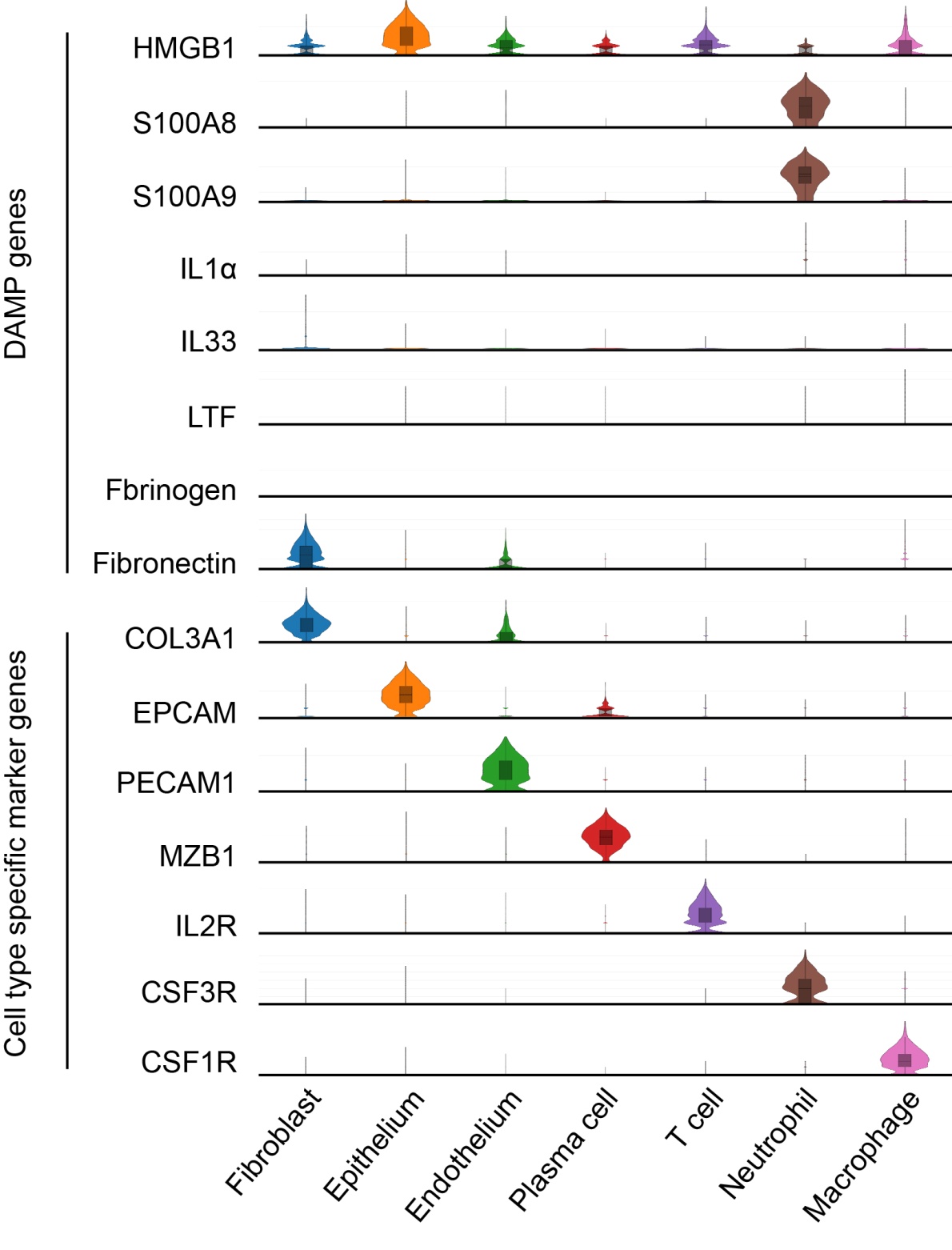
**

**Figure S10. HMGB1 is expressed in intestinal epithelial cells.**

Violin plots showing the smoothened expression distribution of DAMPs (HMGB1, S100A8, S100A9, IL1α, IL33, LTF, Fbrinogen, Fibronectin), and cell type specific marker genes (COL3A1, EPCAM, PECAM1, MZB1, IL2R, CSF3R, CSF1R). Analysis of gene expression in scRNA-seq data (unpublished) was performed using Seurat.
